# Stem cell therapy for Crohn’s disease: systematic review and meta-analysis of preclinical and clinical studies

**DOI:** 10.1186/s13287-021-02533-0

**Published:** 2021-08-18

**Authors:** Ruo Wang, Qigu Yao, Wenyi Chen, Feiqiong Gao, Pan Li, Jian Wu, Jiong Yu, Hongcui Cao

**Affiliations:** 1 State Key Laboratory for the Diagnosis and Treatment of Infectious Diseases, Collaborative Innovation Center for Diagnosis and Treatment of Infectious Diseases, The First Affiliated Hospital, Zhejiang University School of Medicine, 79 Qingchun Rd., Hangzhou City, 310003 China; 2 National Clinical Research Center for Infectious Diseases, 79 Qingchun Rd., Hangzhou City, 310003 China; 3 Zhejiang Provincial Key Laboratory for Diagnosis and Treatment of Aging and Physic-Chemical Injury Diseases, 79 Qingchun Rd., Hangzhou City, 310003 China

**Keywords:** Stem cells, Crohn’s disease, Systematic review and meta-analysis, Crohn’s disease activity index, Histopathological score, Colon length, Remission rate

## Abstract

**Background:**

We explored whether stem cell therapy was effective for animal models and patients with Crohn’s disease (CD).

**Methods:**

We searched five online databases. The relative outcomes were analyzed with the aid of GetData Graph Digitizer 2.26 and Stata 16.0 software. The SYRCLE risk of bias tool and the MINORS tool were used to assess study quality.

**Results:**

We evaluated 46 studies including 28 animal works (*n* = 567) and 18 human trials (*n* = 360). In the animal studies, the disease activity index dramatically decreased in the mesenchymal stem cell (MSC) treatment groups compared to the control group. Rats and mice receiving MSCs exhibited longer colons [mice: standardized mean difference (SMD) 2.84, *P* = 0.000; rats: SMD 1.44, *P* = 0.029], lower histopathological scores (mice: SMD − 4.58, *p* = 0.000; rats: SMD − 1.41, *P* = 0.000) and lower myeloperoxidase levels (SMD − 6.22, *P* = 0.000). In clinical trials, stem cell transplantation reduced the CD activity index (SMD − 2.10, *P* = 0.000), the CD endoscopic index of severity (SMD − 3.40, *P* = 0.000) and simplified endoscopy score for CD (SMD − 1.71, *P* = 0.000) and improved the inflammatory bowel disease questionnaire score (SMD 1.33, *P* = 0.305) compared to control values. CD patients maintained high remission rates for 3–24 months after transplantation.

**Conclusions:**

Stem cell transplantation is a valuable supplementary therapy for CD.

**Supplementary Information:**

The online version contains supplementary material available at 10.1186/s13287-021-02533-0.

## Introduction

Crohn’s disease (CD), a form of inflammatory bowel disease (IBD), is an immune system-mediated, chronic systemic condition characterized by gastrointestinal inflammation and dysregulation of the mucosal-associated immune system [1, 2]. The annual incidence of CD ranges from 5.0 to 20.2 per 100,000 person-years [3, 4], and CD affects more than 1 million people in the USA. The pathogenesis is complex, featuring disturbance of the innate immune system and reduced gastrointestinal barrier protection. Infections and environmental factors may trigger or exacerbate the disease [5]. Corticosteroids (CSs), immunomodulatory agents, and “biological therapies” including anti-TNF-α antibodies are used to suppress intestinal inflammation. However, standard anti-inflammatory regimes do not halt disease progression. Aggressive “biological therapies” are immunogenic, but their effects fade over time [6]. Approximately 25% of CD patients are refractory to such medications and respond to surgery only [7]. It is thus critical to enhance CD remission and reduce recurrence.

In recent years, developments in stem cell (SC) biology and regenerative medicine have revealed that SCs unexpectedly can be used to treat autoimmune diseases. Mesenchymal stem cells (MSCs) and hematopoietic stem cells (HSCs) have been shown to counter rheumatoid arthritis, autoimmune hepatitis, and systemic sclerosis [8]. MSCs exhibit low immunogenicity and immunomodulation. Randomized controlled experiments have shown that local MSCs injection improved CD-related perianal fistulation [9, 10]. HSCs transplantation restored immune tolerance and relieved CD [11]. SCs have been found to inhibit intestinal inflammation, promote long-term intestinal mucosal healing, and significantly improve patient quality of life, making them a valuable alternative CD treatment. Several studies have evaluated the safety and effectiveness of CD stem cell therapy, but the results remain controversial. We thus systematically reviewed the literature and conducted a meta-analysis on the effectiveness and safety of SC therapy.

## Materials and methods

### Search strategy

Five databases (PubMed, Embase, the Web of Science, the Cochrane Library, and Clinical Trials.gov) were searched from their inception dates to February 2021. The search string added the keywords focus on Crohn’s Disease (“Crohn’s Enteritis”, “Regional Enteritis”, “Crohn’s Disease”, “Crohns Disease”, “Inflammatory Bowel Disease 1”, “Enteritis, Granulomatous”, “Granulomatous Enteritis”, “Enteritis, Regional”, “Ileocolitis”, “Colitis, Granulomatous”, “Granulomatous Colitis”, “Ileitis, Terminal”, “Ileitis, Terminal”, “Terminal Ileitis”, “Ileitis, Regional” and “Regional Ileitis”) and stem cells (“stem cells” OR “progenitor cells”, “hematopoietic stem cells”, “mesenchymal stem cells”, “bone marrow mononuclear cells”). We also reviewed secondary references. Two researchers independently screened the titles and abstracts of all retrieved articles.

### Study selection

Studies that met all of the following criteria were included: single-arm studies or randomized controlled trials including CD patients or animal studies; studies featuring SC therapy with no restriction imposed on the type of SCs, route of administration, or dose; and the inclusion of human CD clinical parameters [CD activity index (CDAI) scores, C-reactive protein (CRP) levels, CD endoscopic index of severity (CD-EIS) scores, simplified endoscopy score for CD (SES-CD), inflammatory bowel disease questionnaire (IBDQ) results] or animal disease activity index (DAI) scores, histopathological scores (HSs), colon lengths, myeloperoxidase (MPO) and cytokine levels. Case reports, repeat studies, reviews, and studies lacking full texts were excluded. If more than one article analyzed the same trial, we included only the latest report.

### Data extraction and quality assessment

Two researchers independently evaluated article quality and extracted data by screening abstracts and full texts. A third researcher was consulted to resolve any disagreements. For animal studies, all relevant data were recorded in Microsoft Excel including the first author; year; location; mouse sex, strain, and weight; group numbers; modeling methods; modeling duration; type and source of MSCs; how MSCs were administered; times of treatment; and other parameters. For clinical trials, the following data were recorded: first author, year, location, size of the MSC group, size of the control group, male/female ratio, type of SCs given, number of SCs administered, administration route, times of treatment, treatment course and follow-up duration. The SYRCLE risk of bias tool was used to evaluate the quality of animal studies [12], and the quality of clinical studies was assessed with the aid of the MINORS tool [13]. We adhered to PRISMA guidelines for this systematic review and meta-analysis [14].

### Statistical analysis

The DAI was calculated from clinical parameters of inflammation (weight loss, diarrhea, and rectal bleeding) that reflect CD severity. CD morphological and pathological changes were represented by the colon length and HS. The MPO level reflected the extent of neutrophil infiltration. The standardized mean difference (SMD) with the 95% confidence interval (CI) for each parameter was calculated to reveal changes after stem cell therapy in animals. For data from human studies, the means and standard deviations (SDs) of continuous variables (CDAI, CD-EIS, SES-CD and IBDQ scores and the CRP level) were subjected to SMD analysis. We used odds ratios (ORs) with 95% CIs to determine “remission rates.” Medians with percentiles were converted to means with SDs. If only figures were presented, two researchers independently used GetData Graph Digitizer ver. 2.26 to extract data and compute the means [15]. Among-study heterogeneity was examined using the *I*^2^ test. An *I*^2^ value ≤ 50% indicated homogeneity and a fixed-effect model were employed. An *I*^2^ value > 50% indicated heterogeneity, and a random-effect model was used instead. Subgroup analyses were performed to evaluate heterogeneity. We employed STATA ver. 16.0 to create forest plots and facilitate the meta-analysis. We used the Begg and Egger tests in STATA (with the significance level set to *P* < 0.1) to evaluate publication bias. All tests were two-sided, and *P* < 0.05 was considered to indicate statistical significance.

## Results

### Search results

**Fig. 1:**
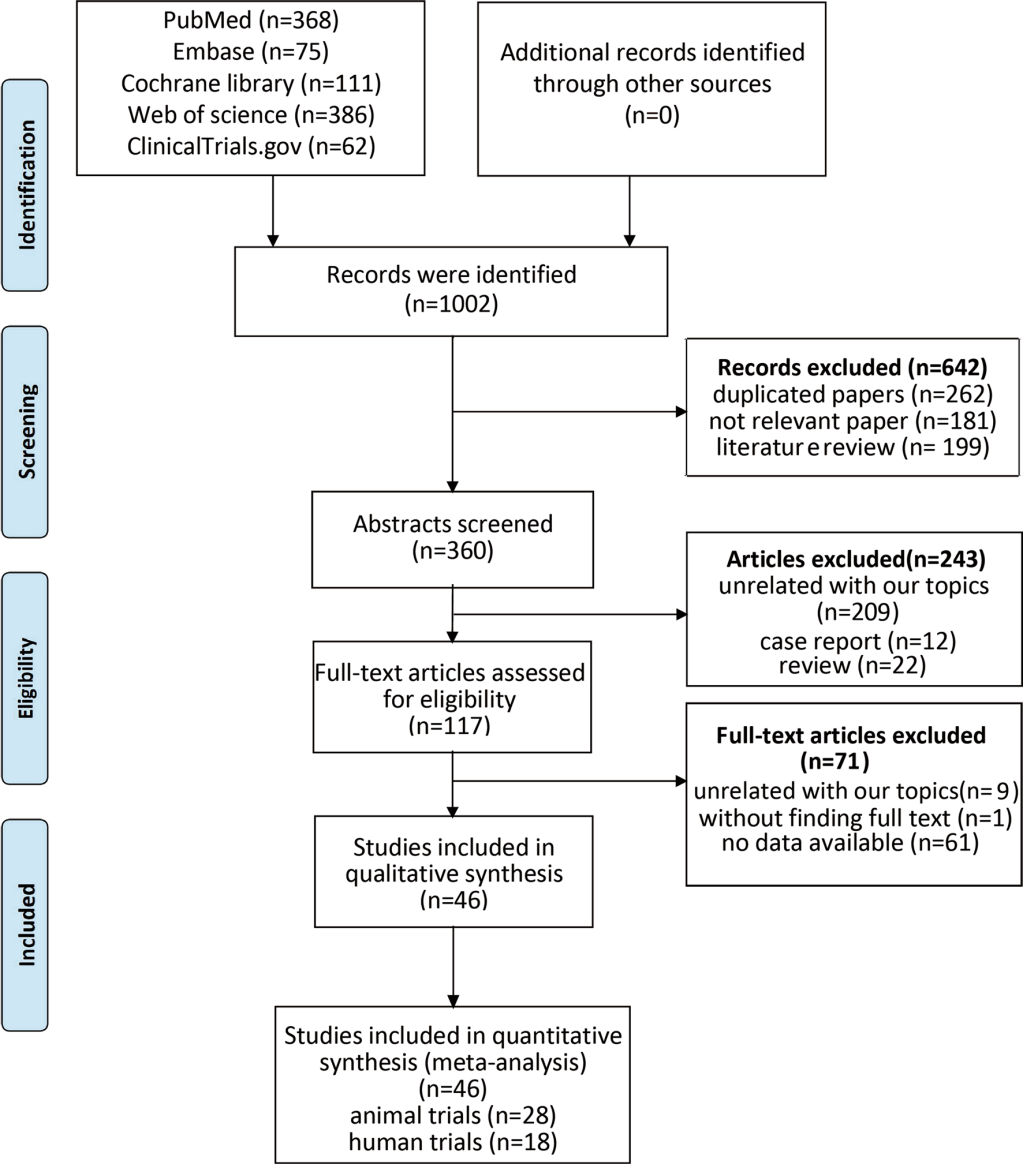
Flowchart of study selection. A total of 1002 records were retrieved; after application of the inclusion criteria, 28 animal studies and 18 human trials remained

A total of 1002 studies were retrieved, from which 262 duplications were initially removed. A review of the titles led to the removal of 380 irrelevant papers; a further 243 were excluded after reading the abstracts. A total of 117 full-text studies were carefully reviewed, of which 61 were excluded for lack of data, 10 because they were off-topic, and 1 because the full text was unavailable. Finally, 28 animal studies [16–43] and 18 human studies [44–61] were selected for the meta-analysis (Fig. 1). Funnel plots were used to evaluate publication bias (Additional file 1: Fig. S1).

### Animal studies

#### Study characteristics and quality

A total of 487 mice and 80 rats were used; 78% of all mice were of the C57BL strain, 19.9% were of the BALB/C strain, and 2.1% were of the NOD.CB17-Prkdcscid/J strain. Of all rats, 20% were of the Wistar strain, 42.5% were of the Sprague–Dawley strain, and 37.5% were of the Lewis strain. All 28 studies used one of two models: either CD groups consumed dextran sodium sulfate (DSS) in water while controls received regular water or colitis was induced by intrarectal administration of trinitrobenzene sulfonic acid (TNBS) in ethanol (controls received ethanol only). Information on study characteristics, study quality, and publication bias is shown in Table 1, Additional file 1: Table S1, and Fig. S1.

**Table 1:** Characteristics of animal experiments

First author Year	Location	Animal (sex, strain, weight)	Number of each group	Modeling method	Modeling duration	Type and source of MSC	Way of MSC administrated	Times of treatment	Dose of MSC	Parameter
Forte; 2015 [16]	Italy	Male, C57BL/6 mice, NA	DSS + PBS (*n* = 4) DSS + MSC (*n* = 4)	DSS (1.5%)	9	hASC	Irrigation	3	1 × 10^6^ cells/mouse/time	DAI, HS
Chao; 2016 [17]	China	Male, BALB/c mice, NA	TNBS + 50% ethanol + MSC (*n* = 20) TNBS + 50% ethanol (*n* = 20)	TNBS (5%)	8	hUC-MSC	Intraperitoneal injection	1	1 × 10^6^ cells/mouse/time	HS, MPO activity
Gao; 2020 [18]	China	Male, Wistar rat, 150–200 g	TNBS + 50% ethanol + MSC (*n* = 8) TNBS + 50% ethanol (*n* = 8)	TNBS (2%)	8	rASC	Tail vein injection	1	1 × 10^7^ cells/mouse/time	DAI, colon length
Nam; 2015 [19]	South Korea	Female, C57BL/6, NA	DSS + PBS (*n* = 10) DSS + MSC (*n* = 10)	DSS (3.5%)	7	mBM-MSC	Intraperitoneal injection	1	1 × 10^6^ cells/mouse/time	DAI
Yang; 2018 [20]	China	Female, C57 mice, 19–21 g	DSS + PBS (*n* = 10) DSS + MSC (*n* = 10)	DSS (1.5%)	6	hUC-MSC	Intraperitoneal injection	2	2 × 10^6^ cells/mouse/time	DAI, HS
Gonzalez-Rey; 2008 [21]	Spain	NA, C57BI/6 mice, NA	DSS + PBS (*n* = 14) DSS + MSC (n = 14)	DSS (5%) DSS (3%)	7 34	hASC	Intraperitoneal injection	1 2	1 × 10^6^ cells/mouse/time	DAI, colon length, HS, MPO activity
Lin; 2015 [22]	China	Male, C57BL/6 mice, NA	DSS + PBS (*n* = 10) DSS + MSC (*n* = 10)	DSS (5%)	7	hUC-MSC	Intraperitoneal injection	1	2 × 10^6^ cells/mouse/time	DAI, colon length, MPO activity
Banerjee; 2015 [23]	Italy	Male, NOD.CB17-Prkdc^scid^/J mice, 18–25 g	DSS + PBS (*n* = 5) DSS + MSC (*n* = 5)	DSS (3.5%)	7	hUC-MSC	Tail vein injection	1	1 × 10^6^ cells/mouse/time	DAI, colon length, HS, MPO activity
Song; 2018 [24]	South Korea	Male, C57BL/6 J mice, NA	DSS + PBS (*n* = 6) DSS + MSC (*n* = 6)	DSS (3%)	7	cAT-MSC	Intraperitoneal injection	1	2 × 10^6^ cells/mouse/time	DAI, colon length, HS
Lim; 2021 [25]	South Korea	Female, C57BL/6 mice, NA	DSS + PBS (*n* = 4) DSS + MSC (*n* = 4)	DSS (2.5%)	9	mBM-MSC	Intraperitoneal injection	1	3 × 10^6^ cells/mouse/time	DAI, colon length
Miyamoto; 2017 [26]	Japan	Male, Sprague–Dawley rats, NA	TNBS + 30% ethanol + MSC (*n* = 9) TNBS + 30% ethanol + PBS (*n* = 9)	TNBS (45 mg/kg)	7	hAMSC	Penile vein injection	1	1 × 10^6^ cells/mouse/time	HS
Ikarashi; 2017 [27]	Japan	Male, C57BL/6 mice, NA	DSS + PBS (*n* = 7) DSS + MSC (*n* = 16)	DSS (2.5%)	7	hASC hUC-MSC	Intravenous injection	1	1 × 10^6^ cells/mouse/time	DAI, colon length, HS
Li; 2020 [28]	China	Female, C57BL/6 mice, 18-22 g	DSS + PBS (*n* = 12) DSS + MSC (*n* = 12)	DSS (3%)	8 24	hUC-MSC	Intraperitoneal injection	2 4	2 × 10^6^ cells/mouse/time	DAI, MPO activity, colon length, HS
Lee; 2016 [29]	South Korea	Female, C57BL/6 mice, 17-21 g	DSS + PBS (*n* = 5) DSS + MSC (*n* = 5)	DSS (3%)	30	mBM-MSC	Intravenous injection	3	1 × 10^7^ cells/mouse/time	Colon length, HS
Jo; 2019 [30]	South Korea	Female, C57BL/6 mice, NA	DSS + PBS (*n* = 5) DSS + MSC (*n* = 5)	DSS (3%)	20	mBM-MSC	Intraperitoneal injection	2	1 × 10^6^ cells/mouse/time	Colon length, HS
Heidari; 2018 [31]	Iran	Female, C57BL/6 mice, 18–22 g	DSS + PBS (*n* = 5) DSS + MSC (*n* = 5)	DSS (2%)	33	mASC	Intraperitoneal injection	2	1 × 10^6^ cells/mouse/time	DAI, colon length, HS
Kawata; 2019 [32]	Japan	Male, C57BL/6 mice, NA	DSS + PBS (*n* = 5) DSS + MSC (*n* = 7)	DSS (2.5%)	7	hASC	Intravenous injection	1	1 × 10^6^ cells/mouse/time	DAI, colon length, HS
Yu; 2017 [33]	South Korea	Male, C57BL/6 mice, 18–25 g	DSS + PBS (*n* = 9) DSS + MSC (*n* = 18)	DSS (1.5%)	30	hTMSC	Intraperitoneal injection	2 4	1 × 10^6^ cells/mouse/time	Colon length
Lu; 2019 [34]	China	NA, C57BL/6 mice, NA	DSS + PBS (*n* = 6) DSS + MSC (*n* = 6)	DSS (3%)	26	hGMSC	Tail vein injection	1	NA	Colon length
Tanaka; 2010 [35]	Japan	NA, Lewis rat, 180–220 g	DSS + PBS (*n* = 10) DSS + MSC (*n* = 10)	DSS (4%)	7	rBM-MSC	Tail vein injection	3	2 × 10^4^ cells/g/time (the second and third injection) 2 × 10^3^ cells/g/time (the first injection)	HS
Molendijk; 2016 [36]	The Netherlands	Female, C57BL/6Jico mice, NA	DSS + PBS (*n* = 7) DSS + MSC (*n* = 21)	DSS (1.25%)	7	mBM-MSC	Local injection	1	0.5 × 10^6^ cells/mouse/time 2 × 10^6^ cells/mouse/time	Colon length
Liang; 2011 [37]	China	Male, BALB/c mice, 20 g	TNBS + 50% ethanol + MSC (*n* = 6) TNBS + 50% ethanol (*n* = 6)	TNBS (100 mg/kg)	5	hUC-MSC	Intravenous injection	2	1 × 10^6^ cells/mouse/time	HS, MPO activity
Fuenzali; 2016 [38]	Chile	Female, C57BL/6 mice, NA	DSS + PBS (*n* = 7) DSS + MSC (*n* = 7)	DSS (2.5%)	7	hUC-MSC	Intraperitoneal injection	2	1 × 10^6^ cells/mouse/time	Colon length
Li; 2013 [39]	China	NA, C57Bl/6 mice, NA	DSS + PBS (*n* = 8) DSS + MSC (*n* = 16)	DSS (4%)	7	hUC-MSC hBM-MSC	Intraperitoneal injection	1	1 × 10^6^ cells/mouse/time	HS, colon length, MPO activity
Tanaka; 2008 [40]	Japan	Male, Lewis rats, approximately 200 g	DSS + PBS (*n* = 5) DSS + MSC (*n* = 5)	DSS (4%)	7	rBM-MSC	Tail vein injection	1	5 × 10^6^ cells/mouse/time	Colon length
Nikolic; 2018 [41]	Serbia	NA, C57BL/6 mice, 19–21 g	DSS + PBS (*n* = 35) DSS + MSC (*n* = 35)	DSS (3%)	7	mBM-MSC	Tail vein injection	3	2 × 10^6^ cells/mouse/time	HS, colon length
Nan; 2018 [42]	China	Male, Sprague–Dawley rats, 160–180 g	TNBS + 50% ethanol + MSC (*n* = 8) TNBS + 50% ethanol (*n* = 8)	TNBS (NA)	7	rBM-MSC	Tail vein injection	1	5 × 10^6^ cells/mouse/time	Colon length, HS
Xie; 2017 [43]	China	NA, BALB/c mice, NA	TNBS + 50% ethanol + MSC (*n* = 30) TNBS + 50% ethanol (*n* = 15)	TNBS (3%)	3	mBM-MSC mASC	Intraperitoneal injection	1	1 × 10^6^ cells/mouse/time	HS

DSS, dextran sodium sulfate; TNBS, trinitrobenzene sulfonic acid; MSC, mesenchymal stem cell; PBS, phosphate buffer saline; hUC-MSC, human umbilical cord MSC; mBM-MSC, murine bone marrow MSC; rBM-MSC, rat bone marrow MSC; hBM-MSC, human bone marrow MSC; hASC, human adipose-derived MSC; mASC, murine adipose-derived MSC; rASC, rat adipose-derived MSC; hTMSC, human tonsil-derived mesenchymal stem cell; hGMSC, human gingiva-derived mesenchymal stem cell; hAMSC, human amnion-derived MSC; cAT-MSC, canine adipose tissue-derived MSC; DAI, disease activity index; HS, histopathological score; MPO, activity myeloperoxidase activity; NA, not available

#### Disease activity index

**Fig. 2:**
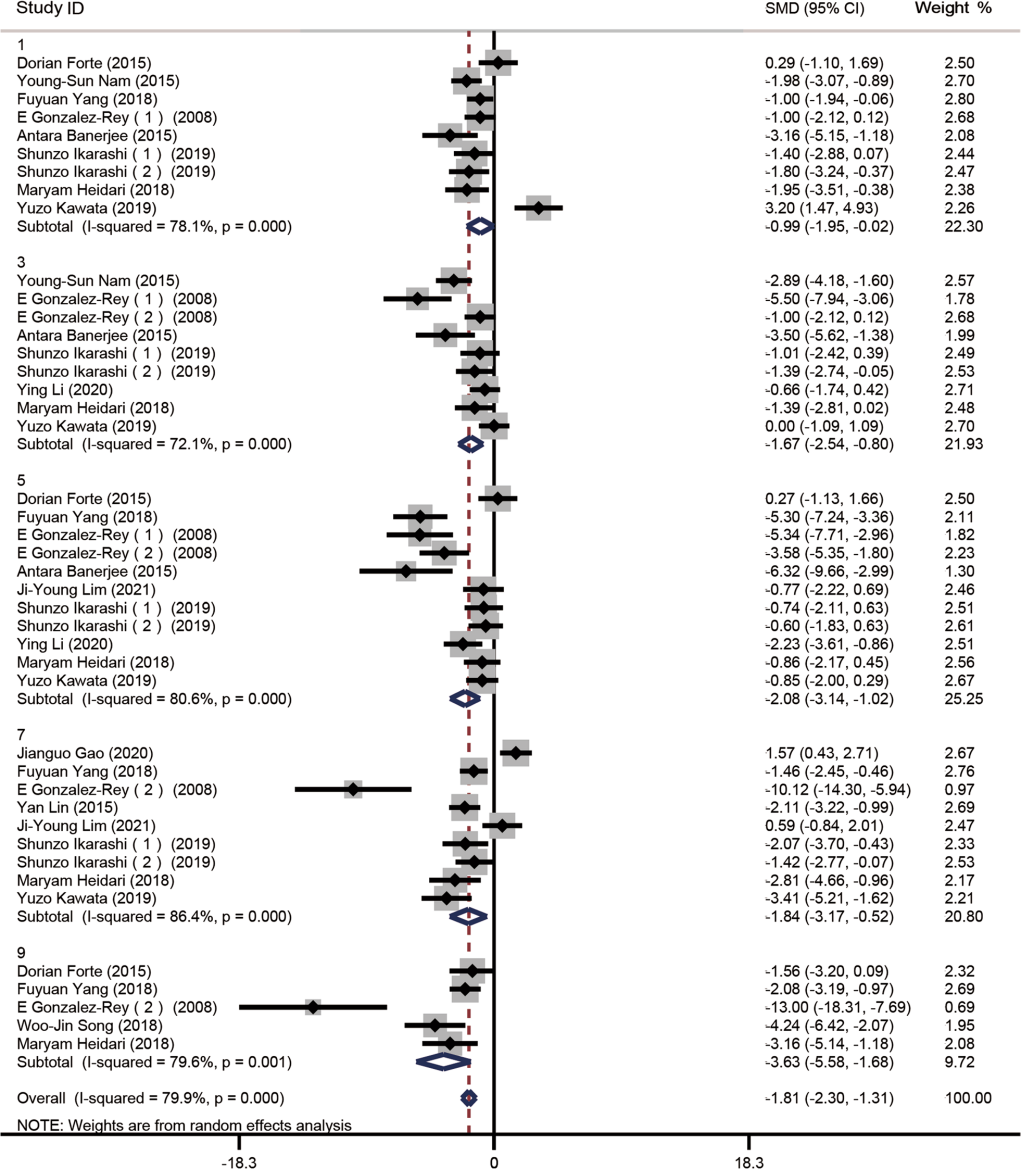
Forest plot of mouse follow-up DAI scores. Compared to the controls, DAI scores decreased in the experimental groups on days 1, 3, 5, 7, 9, and 11 after MSC transplantation

Of the 28 studies, 12 [16, 18–23, 25, 27, 28, 31, 32] reported DAI scores, 9 on day 1 (*n* = 118 animals), 9 on day 3 (*n* = 118), 11 on day 5 (*n* = 134), 3 on day 7 (*n* = 124), and 5 on day 9 (*n* = 64). A random effects model was chosen for analysis, and the Cohen method was used to assess differences in DAI between the treatment and control groups. Subgroup analyses showed that the experimental DAI scores were significantly lower than the control scores on day 1 (SMD − 0.99, 95% CI − 1.95 to − 0.02, *I*^2^ = 78.1%, *P* = 0.000), day 3 (SMD − 1.67, 95% CI − 2.54 to − 0.80, *I*^2^ = 72.1%, *P* = 0.000), day 5 (SMD − 2.08, 95% CI − 3.14 to − 1.02, *I*^2^ = 80.6%, *P* = 0.000), day 7 (SMD − 1.84, 95% CI − 3.17 to − 0.52, *I*^2^ = 86.4%, *P* = 0.000) and day 9 (SMD − 3.63, 95% CI − 5.58 to − 1.68, *I*^2^ = 79.6%, *P* = 0.001) (Fig. 2). Each subgroup exhibited evidence of heterogeneity, which was alleviated in all but the day 5 subgroup after one or two studies were deleted (Additional file 1: Fig. S2 and Fig. S3). The heterogeneity exhibited by the day 3 and 9 subgroups fell to moderate levels after deleting the studies of Banerjee et al. [23] and Gonzalez-Rey et al. [21]; and Gonzalez-Rey et al. [21], respectively. The heterogeneity associated with the day 1 and 7 subgroup disappeared after excluding Kawata et al. [32] and Forte et al. [16]; Gao et al. [18], Gonzalez-Rey et al. [21] and Ji Young Lim et al. [26], respectively.

#### Colon length

**Fig. 3:**
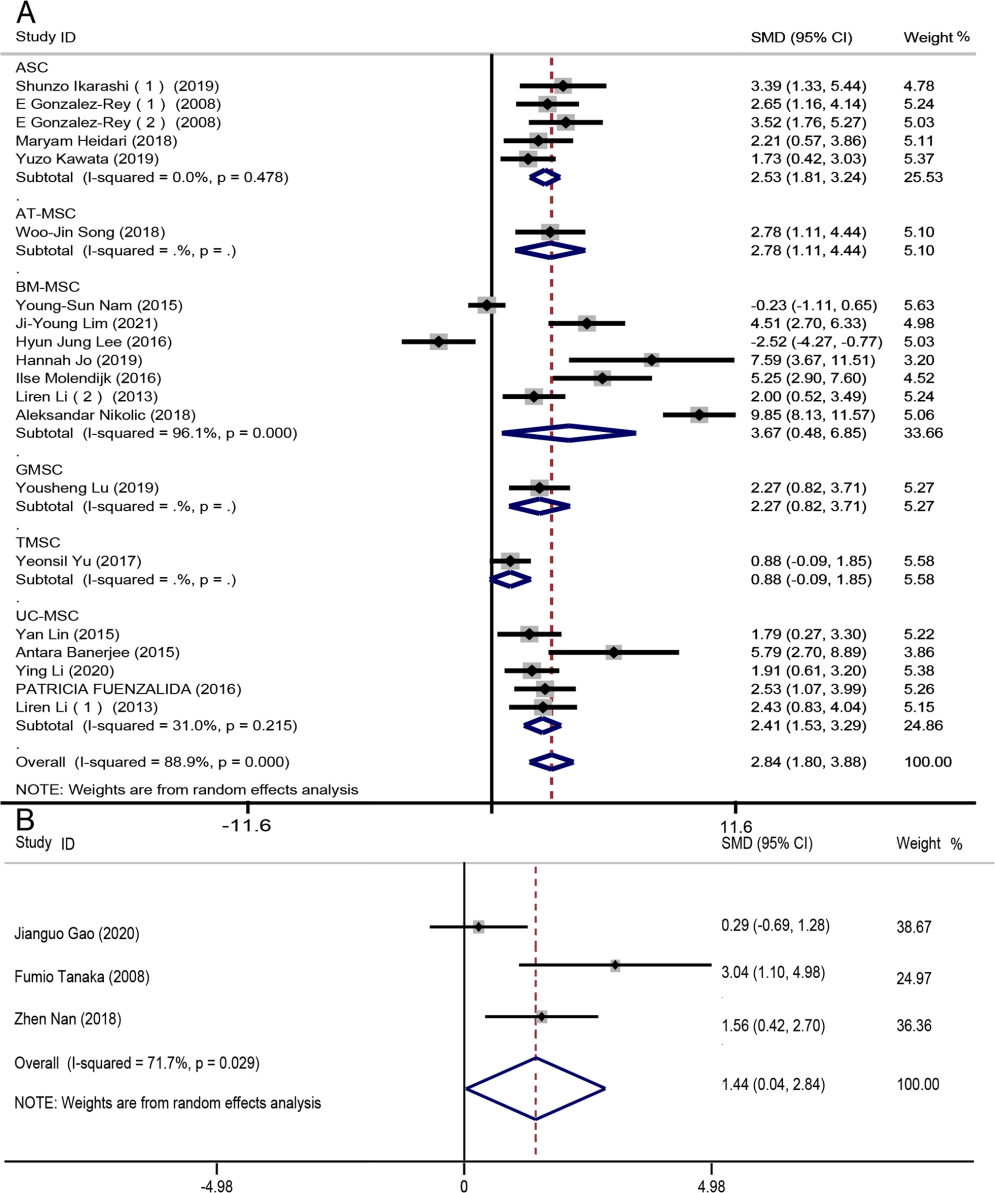
Forest plots of subgroup analyses by colon length and MSC type for **a** mouse groups and **b** rat groups. After treatment, the colon lengths in the experimental groups were longer than those in the controls, indicating that colitis was alleviated. All mouse subgroups (except the BM-MSC subgroup) exhibited low-level heterogeneity

As animal colon lengths differ, we evaluated mice and rat data separately. 18 studies [19, 21–26, 28–34, 36, 38, 39, 41] described mouse colon lengths (*n* = 338 mice). We used a random-effects model to compare colon lengths between treatment and control groups employing the Cohen method. Colon lengths increased markedly in the experimental groups (SMD 2.84, 95% CI 1.80 to 3.88, *I*^2^ = 87.688.9%, *P* = 0.000). As the *I*^2^ value was high, we performed subgroup analysis by MSC type. Subgroup heterogeneity was low except in the bone marrow-derived MSC (BM-MSC) group (Fig. 3a).

Three studies [18, 41, 43] (*n* = 42 animals) reported rat colon lengths; we again used a random-effect model to compare colon lengths between the treatment and control groups while employing the Cohen method. Colon lengths in the experimental groups were longer than those in the control groups (SMD 1.44, 95% CI 0.04–2.84, *I*^2^ = 71.7%, *P* = 0.029) (Fig. 3b). Heterogeneity was high; sensitivity analysis showed that this was explained by the work of Gao et al. [18]. After this was excluded, the level of heterogeneity decreased (*I*^2^ = 40.2%, *P* = 0.003) (Additional file 1: Fig. S4).

#### Histopathological scores

**Fig. 4:**
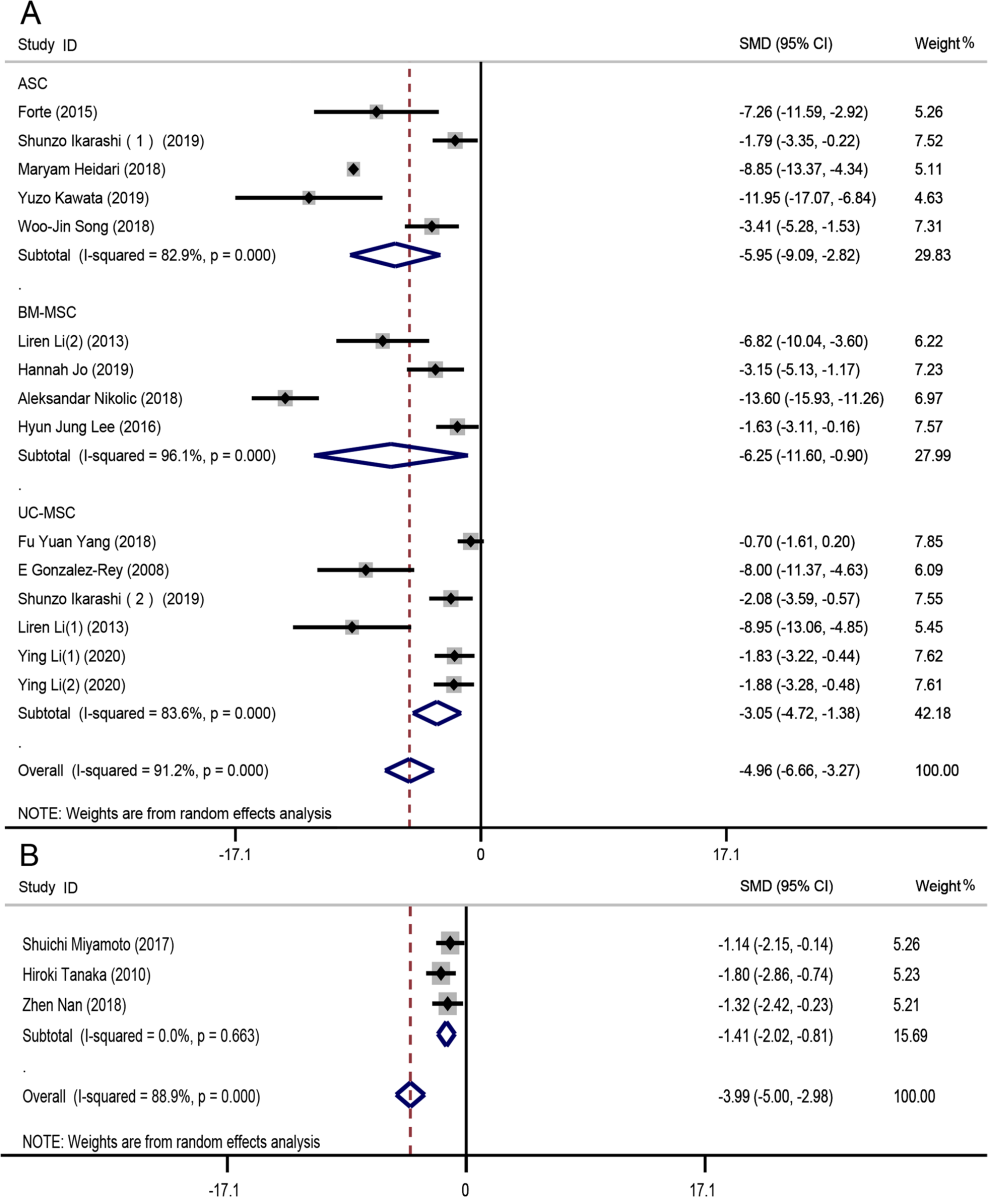
Forest plot of histopathological score (HS) level. **a** Forest plot of the HSs in mouse groups undergoing MSC transplantation and control groups. **b** Forest plot of rat HSs after MSC transplantation. The HSs decreased significantly after MSC transplantation in both DSS-induced CD mouse and rat groups

In mice, compared to the control groups, the HS decreased significantly after transplantation (SMD − 4.58, 95% CI − 5.80 to − 3.35, *I*^2^ = 89.6%, *P* = 0.000). Subgroup analysis by the model used showed that the HS for the DSS group (SMD − 4.96, 95% CI − 6.66 to − 3.27, *I*^2^ = 91.2%, *P* = 0.000) was lower than that for the TNBS group (SMD − 3.76, 95% CI − 5.45 to − 2.06, *I*^2^ = 83.8%, *P* = 0.000) (Additional file 1: Fig. S5). Both subgroups exhibited evidence of high heterogeneity. Subgroup analysis by MSC type in the DSS group indicated that BM-MSCs (SMD − 6.25, 95% CI − 11.60 to − 0.90, *I*^2^ = 96.1%, *P* = 0.000) imparted better effects than did other MSC types (Fig. 4a). The heterogeneity observed in the subgroup analyses was attributable to the studies of Ikarashi et al. [27] and Song et al. [24] in the adipose-derived MSC subgroup, Nikolic et al. [41] in the BM-MSC subgroup, and Gonzalez-Rey et al. [21] and Li et al. [39] in the umbilical cord-derived MSC (UC-MSC) subgroup. After excluding these studies, the level of heterogeneity decreased in the adipose-derived MSC subgroup (*I*^2^ = 0), BM-MSC subgroup (*I*^2^ = 76.5%), and UC-MSC subgroup (*I*^2^ = 22.6%) (Additional file 1: Fig. S6). The subgroup analysis of TNBS group decreased the heterogeneity to a lower level (Additional file 1: Fig. S7A).

For the three rat studies [26, 35, 42] (*n* = 54 animals), the HS was lower in the experimental groups than in the control groups (SMD − 1.41, 95% CI − 2.02 to − 0.81, *I*^2^ = 0, *P* = 0.000) and heterogeneity was lacking (Fig. 4b).

#### Myeloperoxidase level

Six studies [17, 21–23, 28] reported MPO levels (*n* = 102 animals). The meta-analysis indicated that the MPO level in the treatment groups was significantly lower than that in the control groups (SMD − 6.22, 95% CI − 9.20 to − 3.32, *I*^2^ = 90.8%, *P* = 0.000) (Additional file 1: Fig. S7B). The level of heterogeneity could not be reduced by excluding any one or two studies.

#### Cytokine level

21 studies showed the level of cytokines before and after the treatment [16–19, 21, 22, 24–27, 29–33, 36, 37, 39, 40, 42, 43]. In both mRNA and protein level, there was a dramatic decrease in IL-6 [mRNA(GAPDH): SMD − 1.18, 95% CI − 1.77 to − 0.60, *I*^2^ = 0, *P* = 0.000; protein (colon): SMD − 3.75, 95% CI − 5.18 to − 2.33, *I*^2^ = 77%, *P* = 0.000], IL-17 [mRNA(GAPDH): SMD − 0.66, 95% CI − 1.21 to − 0.11, *I*^2^ = 0, *P* = 0.000; protein (colon): SMD − 3.51, 95% CI − 5.44 to − 1.58, *I*^2^ = 76%, *P* = 0.000] and TNF-α [mRNA (GAPDH): SMD − 0.88, 95% CI − 1.54 to − 0.23, *I*^2^ = 34.3%, *P* = 0.000; protein (colon): SMD − 2.24, 95% CI − 3.04 to − 1.43, *I*^2^ = 5%, *P* = 0.000] compared with the control group (Additional file 1: Fig. S8 and Fig, S9). IL-10, which is an anti-inflammatory cytokine, increased after the treatment in mRNA level [mRNA (β-actin): SMD 4.30, 95% CI 2.17–6.43, *I*^2^ = 84.7%, *P* = 0.000] (Additional file 1: Fig. S8–S9).

### Human studies

#### Description and quality assessment

We evaluated 18 human studies including 360 patients. The clinical characteristics, results of quality evaluation, and patient demographics are summarized in Table 2.

**Table 2:** Characteristics of clinical trials

First author; year	Location	Number of experimental group	Number of control group	Male/female	Type and source of stem cells	Way of stem cells administrated	Doses of stem cells	Times of treatment	Treatment course	Time of following-up	MINORS
Liang; 2012 [44]	China	4	NA	3/1	Allogeneic MSC	Intravenous infusions	1 × 10^6^/kg body weight	1	N/A	3 months	8
Hasselblatt; 2012 [45]	Germany	8	NA	7/1	Autologous HSC	Intravenous infusions	5.78 × 10^6^/kg body weight	1	Mobilization: 17.8 days Transplantation: 9.8 days	3.1 years	11
Dhere; 2016 [46]	USA	12	4	6/6	Autologous BM-MSC	Intravenous infusions	2 × 10^6^, 5 × 10^6^, 10 × 10^6^/kg body weight	1	N/A	9 weeks	13
Duijvestein; 2010 [47]	The Netherlands	7	NA	1/6	Autologous BM-MSC	Intravenous infusions	1–2 × 10^6^/kg body weight	2	8 days	14 weeks	12
Lopez-Garcia; 2017 [48]	Spain	22	NA	8/14	Autologous HSC	Intravenous infusions	10 × 10^6^/kg body weight	1	Mobilization: 22 days Transplantation: 27 days	12 months	12
Burt; 2010 [49]	USA	24	NA	12/12	Autologous HSC	Intravenous infusions	6.35 × 10^6^/kg body weight	1	Transplantation: 11 days	5 years	12
Zhang; 2018 [50]	China	41	41	24/17	Allogeneic UC-MSC	Intravenous infusions	1 × 10^6^/kg body weight	4	4 weeks	12 months	20
Melmed; 2015 [51]	USA	50	16	NA	PDA-001	Intravenous infusions	1.5 × 10^8^, 6 × 10^8^, 12 × 10^8^	2	8 days	24 months	20
Jauregui-Amezaga; 2015 [52]	Spain	26	NA	18/8	Autologous HSC	Intravenous infusions	14.6 × 10^6^/kg body weight	1	Mobilisation: 18.5 days Transplantation: 26 days	12 months	10
Forbes; 2014 [53]	Australia	15	NA	6/9	Allogeneic BM-MSC	Intravenous infusions	2 × 10^6^/kg body weight	4	3 weeks	42 days	11
Hawkey; 2015 [54]	UK	23	NA	10/13	Autologous HSC	Intravenous infusions	9 × 10^6^/kg body weight	1	NA	12 months	22
Mayer; 2014 [55]	USA	12	NA	3/9	PDA-001	Intravenous infusions	2 × 10^8^, 8 × 10^8^	2	8 days	12 months	12
Gregoire; 2018 [56]	Belgium	13	NA	4/9	Allogeneic BM-MSC	Intravenous infusions	1.5–2.0 × 10^6^/kg body weight	2	4 weeks	12 weeks	12
Cassinotti; 2008 [57]	Italy	4	NA	3/1	Autologous HSC	Intravenous infusions	11 × 10^6^/kg body weight	1	Mobilization: NA Transplantation: 24.5 days	12 months	12
Snowden; 2014 [58]	UK	6	NA	3/3	Autologous HSC	Intravenous infusions	NA	1	NA	87 months	9
Ruiz; 2017 [59]	Brazil	14	NA	7/7	Autologous HSC	Intravenous infusions	13.4 × 10^6^/kg body weight	1	NA	1 month	11
Oyama; 2005 [60]	USA	12	NA	6/6	Autologous HSC	Intravenous infusions	7.7 × 10^6^/kg body weight	1	Mobilization: NA Transplantation: 11 days	18.5 month	11
Clerici; 2011 [61]	Italy	6	NA	2/4	Autologous HSC	Intravenous infusions	10.9 × 10^6^/kg body weight	1	Mobilization: 9 days Transplantation: 13 days	12 months	11

MSC, mesenchymal stem cells; HSC, hematopoietic stem cell; PDA-001, a preparation of mesenchymal-like adherent cells derived from postpartum placentas; MINORS, methodological index for non-randomized studies; NA, not available; N/A not applicable

#### Crohn’s disease activity index

**Fig. 5:**
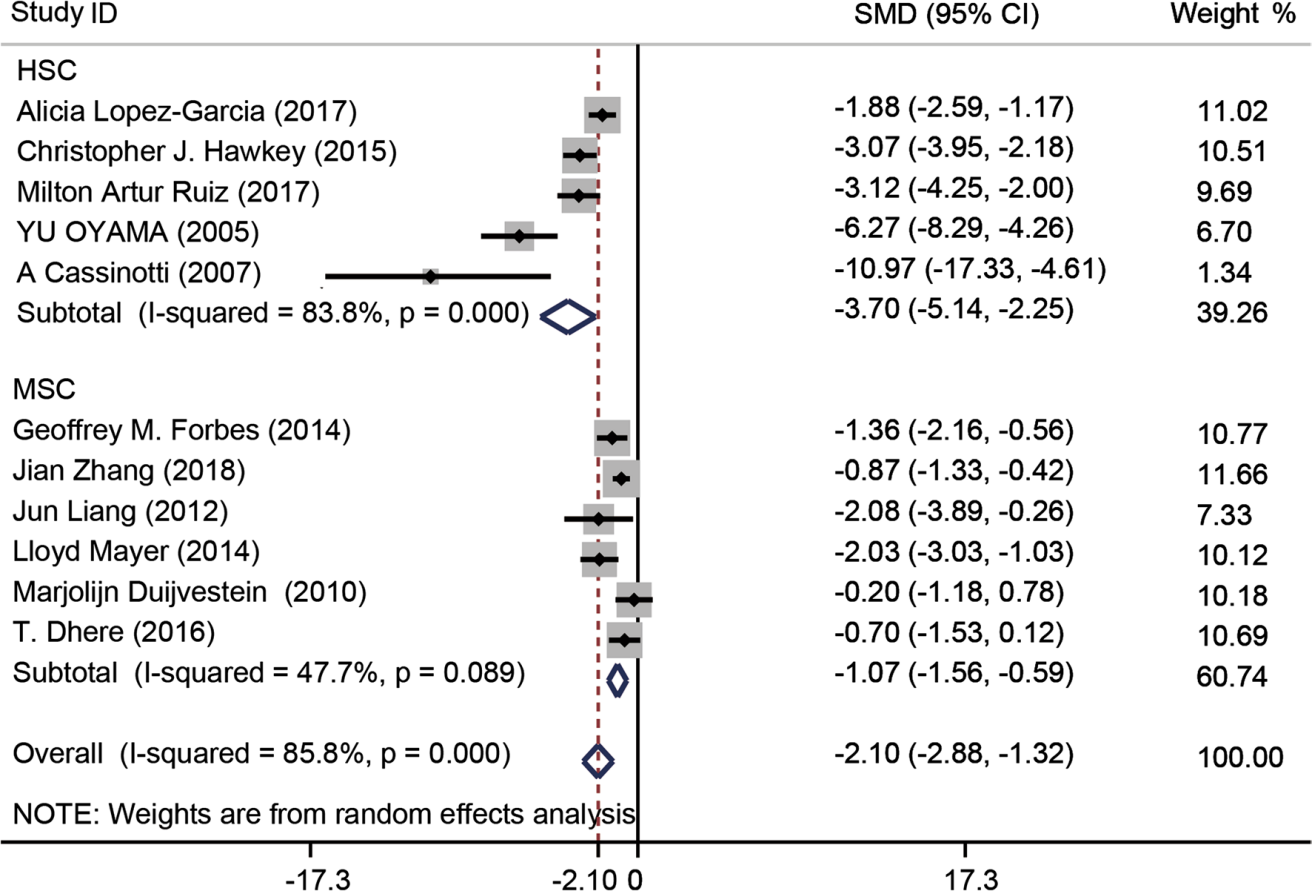
Forest plot of Crohn’s disease activity index (CDAI) scores for clinical trial. Subgroup analysis by stem cell treatment type revealed that the CDAI score decreased after treatment

Eleven [44, 46–48, 50, 53–55, 57, 59, 60] of the 18 studies included CDAI scores, which were significantly lower in the transplantation groups compared to the control groups (SMD − 2.10, 95% CI − 2.88 to − 1.32, *I*^2^ = 85.8%, *P* = 0.000). Subgroup analysis by stem cell type revealed that HSCs (SMD − 3.70, 95% CI − 5.14 to − 2.25, *I*^2^ = 83.8%, *P* = 0.000) afforded more stable outcomes than did MSCs (SMD − 1.07, 95% CI − 1.56 to − 0.59, *I*^2^ = 47.7%, *P* = 0.000) (Fig. 5). After excluding the studies of Oyama et al. [60] and Cassinotti et al. [57], *I*^2^ decreased from 83.6% to 64.6% (Additional file 1: Fig. S10A). In addition, the subgroup analysis by the source of stem cells indicated that the effect of autologous stem cells (SMD − 2.42, 95% CI − 3.51 to − 1.33, *I*^2^ = 88%, *P* = 0.000) was better than the allogeneic stem cells (SMD − 1.48, 95% CI − 2.41 to − 0.55, *I*^2^ = 62.9%, *P* = 0.000) (Additional file 1: Fig. S10B). The stem cells were injected for different times in the studies we evaluated. In order to find the better treatment times, we did another subgroup analysis focus on the times of treatment. The result showed that the CDAI score of groups injected once (SMD − 2.96, 95% CI − 4.19 to − 1.73, *I*^2^ = 85.9%, *P* = 0.000) decreased more than other groups which was injected stem cells twice or fourth times (Additional file 1: Fig. S11).

#### Remission rates

**Fig. 6:**
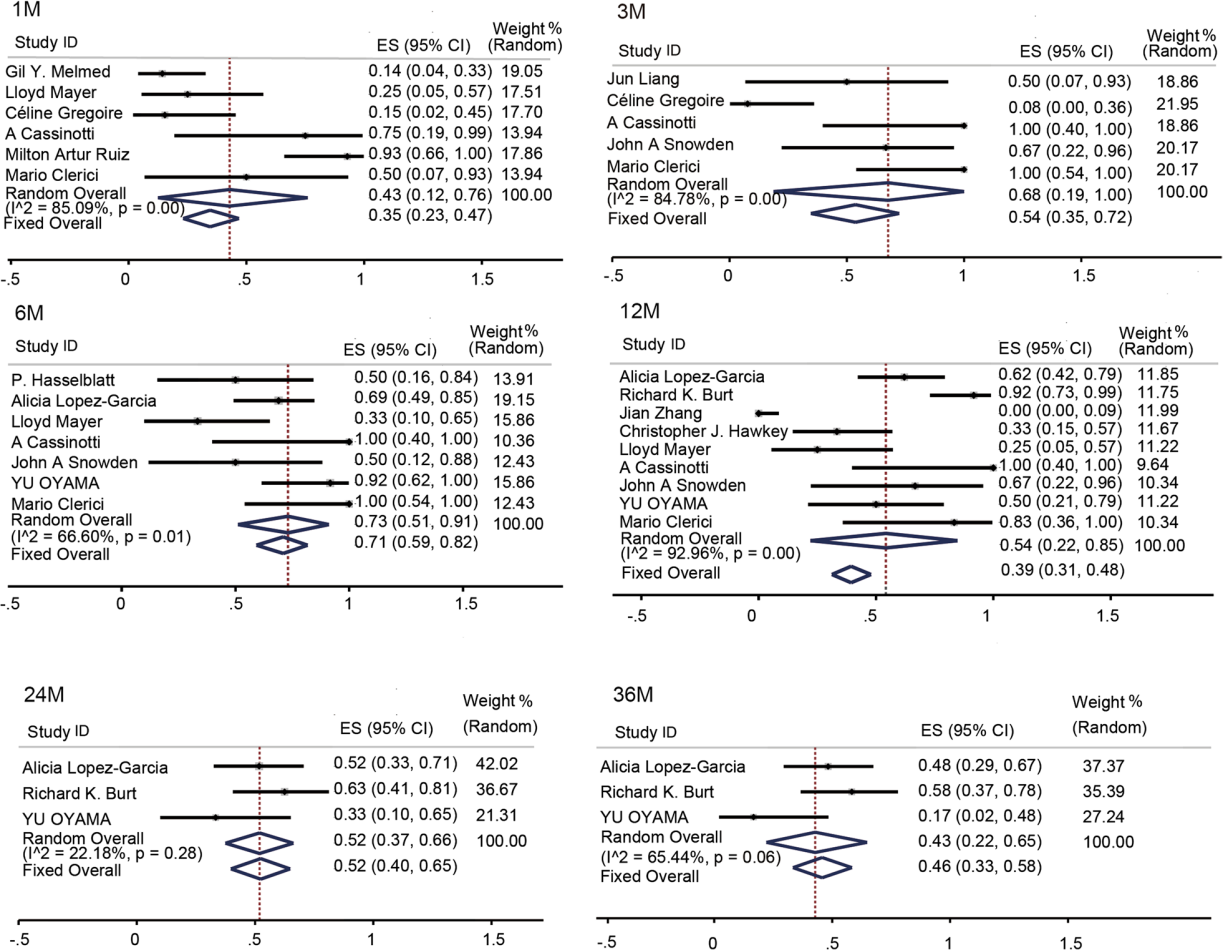
Forest plots of subgroup remission rates by time. The remission rates at 1, 3, 5, 12, 24, and 36 months after st em cell therapy were 43%, 68%, 73%, 54%, 52%, and 46%, respectively, thus both high and stable

Clinically, a CDAI < 150 is defined as indicating remission. Fourteen studies [44, 45, 49–52, 54–61] reported the numbers of patients in remission after treatment. The remission rates at 1, 3, 6, 12, 24, and 36 months after transplantation were 43% (95% CI 0.12–0.76, *I*^2^ = 85.09%, *P* = 0.000), 68% (95% CI 0.19–1, *I*^2^ = 84.78%, *P* = 0.000), 73% (95% CI 0.51–0.91, *I*^2^ = 66.6%, *P* = 0.000), 54% (95% CI 0.22–0.85, *I*^2^ = 92. 96%, *P* = 0.000), 52% (95% CI 0.37–0.66, *I*^2^ = 22.18%, *P* = 0.000), and 43% (95% CI 0.22–0.65, *I*^2^ = 65.44%, *P* = 0.000), respectively (Fig. 6).

#### Endoscopic sore

**Fig. 7:**
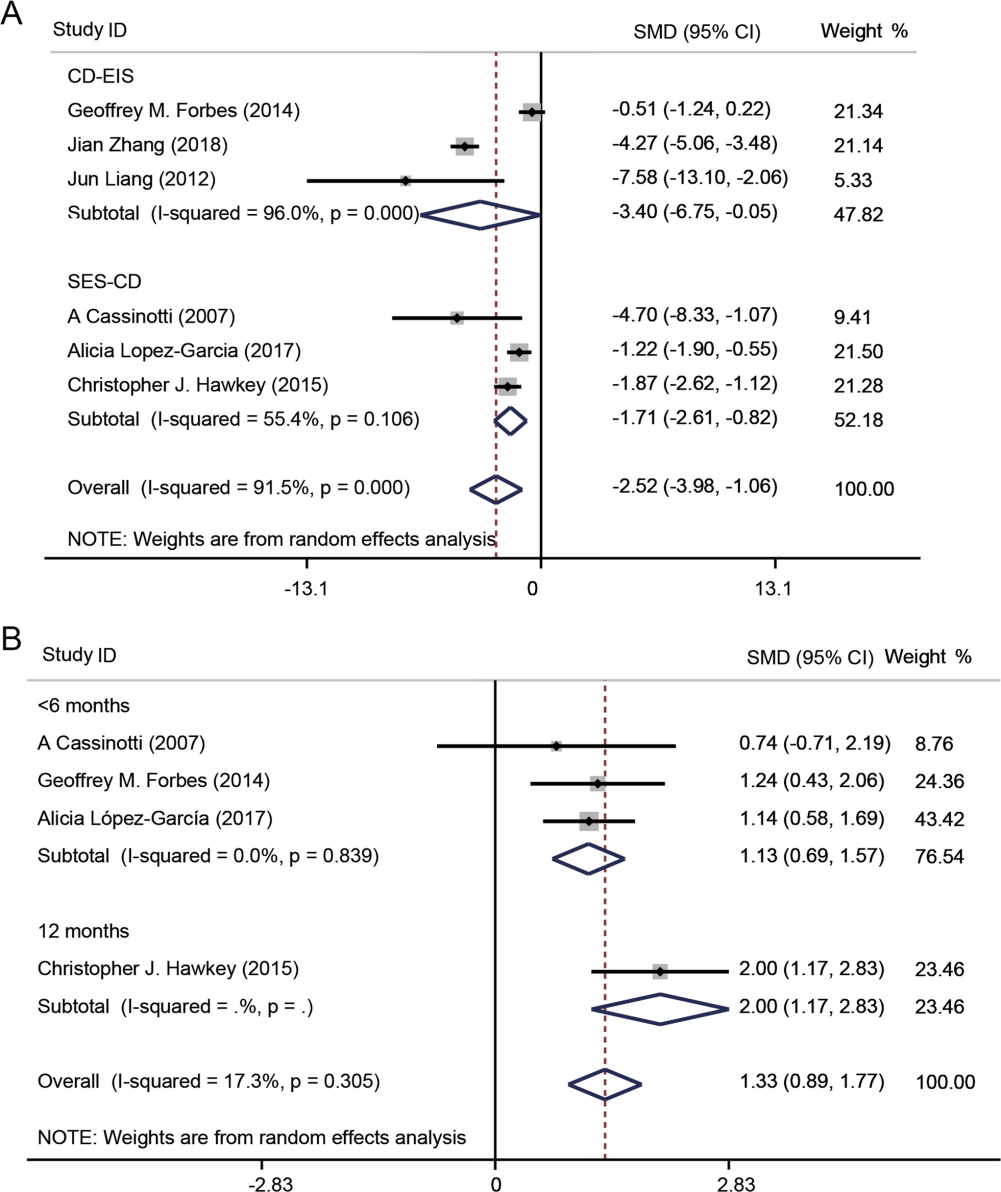
Forest plots of Crohn’s disease endoscopic index of severity (CD-EIS), simplified endoscopy score for CD (SES-CD) and inflammatory bowel disease questionnaire (IBDQ). **a** Forest plots of CD-EIS and SES-CD. After the SC treatment, CD-EIS and SES-CD were lower than the pretreatment score. **b** Forest plots of subgroup IBDQ by time. The IBDQ score after stem cell treatment increased compare with the score before stem cell transplantation

In clinical, CD-EIS and SES-CD were always used to access endoscopic activity of CD patients. Three studies [44, 51, 54] reported CD-EIS data, and another three studies [48, 54, 57] used SES-CD to access the endoscopic remission. The CD-EIS scores for the cell transplantation groups were lower than the pretreatment scores (SMD − 3.40, 95% CI − 6.75 to − 0.05, *I*^2^ = 96%, *P* = 0.000). In the same way, the SES-CD score of people who received stem cell treatment reduced compared with the score before treatment (SMD − 1.71, 95% CI − 2.61 to − 0.82, *I*^2^ = 55.4%, *P* = 0.000) (Fig. 7a).

#### Quality of life

The IBDQ is always used to assess the quality of life of CD patients. Higher score means better quality of life. Four studies [48, 53, 54, 57] provided such data. After transplantation, the quality of life improved (SMD 1.33, 95% CI 0.89–1.77, *I*^2^ = 17.3%, *P* = 0.305). The subgroup analysis showed that the groups injected HSC (SMD 1.37, 95% CI 0.70–2.03, *I*^2^ = 44.2%, *P* = 0.000) had a higher IBDQ score than the groups received BM-MSC treatment (Additional file 1: Fig. S12A). In addition, it was indicated that after the therapy for 12 months, the IBDQ score increased compared with the score accessed after the treatment less than 6 months (Fig. 7b).

#### Laboratory tests

CRP levels are acutely elevated during infection or inflammation. Five studies [53–56, 60] reported CRP data; the levels did not differ greatly before and after treatment (SMD − 0.06, 95% CI − 0.39 to 0.27, *I*^2^ = 0, *P* = 0.988) (Additional file 1: Fig. S12B).

#### Adverse events, complications and recurrence

430 cases of adverse effects were reported in 18 studies, and there were 3 studies [46, 50, 54] showing the data about the number of the adverse events in both experimental groups and placebo groups. The heterogeneity was low across each trial (*I*^2^ = 45.8%, *P* = 0.16) (Additional file 1: Fig. S12C). Common adverse events included viral infections, fever, neutropenia, adrenal insufficiency, and headache. After HSC transplantation, the main adverse reaction was infection during HSC mobilization and regulation. Two patients died of cytomegalovirus infections. Thus, during HSC mobilization, the drug doses prescribed and patient care are critical. In the MSC transplantation group , the main adverse reactions were fever and headache, which were mild and often self-healing. One patient developed well-differentiated stage I sigmoid colon adenocarcinoma; active enteritis had discouraged exploratory endoscopy.

A total of 7 studies (*n* = 82) reported the recurrence data and relapses occurred in 29 patients. There was 10 relapsing within one year after the treatment, and the rest 3, 15 and 1 patients presented clinical and/or endoscopic relapse after 12 months, 53.1 weeks and 15 months after transplant, respectively.

## Discussion

CD is usually treated by addressing the symptoms. However, many patients relapse, and the preferred drugs can have very serious side effects. An effective and safe treatment is urgently required. We explored whether SCs could be used to treat CD in animals and human. SCs reduced intestinal inflammation, enhanced (endoscopically evaluated) mucosal healing, and improved the quality of life in CD patients. SC transplantation should be recommended in clinical practice.

After subgroup and sensitivity analyses, heterogeneity among studies remained very high, attributable to the injection method and model used as well as differences in stem cell types. Of 28 animal studies, 14 featured intraperitoneal injections, 7 tail vein injections, and the rest other injections. Two different mouse CD models (DSS and TNBS) were employed; DSS concentrations ranged from 1.25 to 5% (w/v), and the model duration ranged from 3 to 34 days. Finally, SC donor age, health status, whether the cells were frozen, endpoints, and whether the cells came from the same species as the recipient may all impact the therapeutic effect. More high-quality clinical and animal trials are required.

We compared the responses of animals with DSS- and TNBS-induced CD to MSC treatment. MSCs were therapeutic in both models, but more so in the DSS model. Similarly, in mice with CD, BM-MSCs had a stronger therapeutic effect than did other MSCs.

The safety of SC therapy requires attention. We found that the HSC treatment group was more prone to adverse reactions such as viral infections, which often accompany HSC mobilization and regulation. It is clear that CD patients are at higher risk of infection compared to those who undergo transplantation to treat cancer or other diseases that do not involve the intestinal tract. During SC mobilization, patient immunity is reduced and the risk of infection is higher. Therefore, patients should be carefully nursed during mobilization and reasonable drug levels should be prescribed to reduce the development of adverse reactions. To prevent complications in CD patients with perianal disease, it is advisable to perform drainage, implement strict hygiene measures for contact, and prescribe adequate antibiotic prophylaxis [62]. The implementation of such measures in recent studies dramatically improved safety [52]. MSCs derived from bone marrow or the umbilical cord were associated with lower risks of infection, and most side effects were mild and not associated with MSC injection. UC-MSCs are obtained easily and less invasively, as the donors are young [63], their cell status is good, and the immunogenicity is low. In summary, both treatment efficacy and the type of therapy require attention.

However, the pathogenesis of CD is complex and remains poorly understood [64]. The western diet has been suggested to contribute to the rising incidence of inflammatory bowel diseases. A recent study reported that the interaction between fructose and its transporter, GLUT5, could shape the colonic microbiota and then impact the severity of CD [65]. Another studies showed that serum exosomes could circulate into the intestinal mucosa, regulating macrophage activation and epithelial barrier function to aggravate colitis [66]. Additionally, immune system disorders are clearly in play. Researchers found that advanced oxidation protein products (AOPPs), mainly deposited in macrophages of CD patients, induced macrophage’s lysosomal dysfunction and M1 polarization, which could lead to the intestinal inflammation [67]. CD4^+^ T cells are involved in CD initiation and development, and Th1 or Th2 cells are involved in inflammation [68–70]. The levels of mucosal CD4^+^ helper T cells that secrete effector cytokines such as TNF-α and IFN-γ are abnormally high in the guts of CD patients [71]. Immunomodulatory mechanisms are constrained in such patients. For example, the numbers of immunosuppressive regulatory T cells are significantly reduced in CD patients [72]. The development and maintenance of intestinal inflammation in CD patients probably reflect an imbalance between pro- and anti-inflammatory mechanisms. MSC secrete growth factors, exosomes, cytokines, and metabolites that inhibit inflammation, restore the intestinal mucosal barrier, and are protective. HSCs regenerate self-tolerant lymphocytes in non-inflammatory environments after conditioning that induces an immediate immune cease-fire [73].

Besides MSCs, cytokines and extracellular vehicles (EVs) which are released by MSC also have the therapeutic effect on CD. Because of the low immunogenic profile, which decreases the potential for cell rejection and graft-versus-host-disease, more and more studies begin to focus on the paracrine action of MSC. Neda Heidari et al. [74] and Ju-Hyun An et al. [75] showed that exosomes and prostaglandin E2, isolated from adipose-derived MSC (ASC), could regulate the Treg population and improve acute colitis inflammation induced by DSS.

Stem cell therapy is not only useful for refractory luminal CD, but also shows good effect on the complications of CD [76]. About 20% of CD patients develop to perianal fistulizing Crohn’s disease (pCD), and it is easy to recur [77]. Cx601 (darvadstrocel) is a suspension of human allogenic ASC and its indication is the complex pCD [78]. A lot of clinical trials have proved the effectiveness of treatment for pCD [79–81]. The strong evidence was mainly from a randomized, double-blind, placebo-controlled, multicenter trial (ADMIRE-CD), which indicated that patients who received ASC transplantation had a higher rate of combined remission than the placebo group.

Our work had certain limitations. First, most human studies were single-arm trials with few patients and thus of low quality. Second, CD-EIS, SES-CD data (which reflect endoscopic mucosal healing), IBDQ scores (which reflect the quality of life) and pathological grading were lacked in some studies. Any role for SCs in CD alleviation requires further evaluation.

## Conclusion

We carefully reviewed whether SC therapy improved CD. Stem cell transplantation reduced gut inflammation and improved the quality of life. However, more high-quality randomized controlled clinical trials and basic research are required.

## Supplementary Information

**Additional file 1:Table S1:** The study quality of animal studies; **Figure S1:** Publication bias of the outcomes: histopathological score, colon length, MPO activity, CDAI; **Figure S2:** The sensitivity analysis about DAI scores of the 1st day and the 3rd day after the treatment; **Figure S3:** The sensitivity analysis about DAI score of the 7th day and the 9th day after the treatment; **Figure S4:** The sensitivity analysis about colon length in the rat group; **Figure S5:** The subgroup analysis of histopathological score in the mouse group about modeling methods; **Figure S6:** The sensitivity analysis of histopathological score in the DSS mouse model group; **Figure S7:** The sensitivity analysis of histopathological score in the TNBS mouse model and the forest plot about the level of MPO activity of animal studies. **Figure S8:** The forest plot of IL-6, and IL-17 in the both mRNA and protein levels. **Figure S9:** The forest plot of IL-10 and TNF-α in the both mRNA and protein levels; **Figure S10:** The sensitivity analysis of HSC subgroup and subgroup analysis of CDAI score of different stem cell sources. **Figure S11:** The sensitivity analysis of CDAI scores of different treatment times; **Figure S12:** The subgroup analysis of IBDQ scores of different stem cell types, the forest plots of CRP and the adverse events happened in the experimental and placebo groups.

## Data Availability

All data generated or analyzed during this study are included in this article.

## Abbreviations

CD: Crohn’s disease
SMD: Standardized mean difference
ORs: Odds ratios
CI: Confidence interval
SD: Standard deviation
PRISMA-P: Preferred reporting items for systematic review and meta-analysis protocols
DSS: Dextran sulfate sodium
TNBS: Trinitrobenzene sulfonic acid
DAI: Disease activity index
HS: Histopathological score
MPO: Myeloperoxidase
MSC: Mesenchymal stem cell
BM-MSC: Bone marrow mesenchymal stem cell
UC-MSC: Umbilical cord mesenchymal stem cell
ASC: Adipose-derived mesenchymal stem cell
HSC: Hematopoietic stem cell
IBD: Inflammatory bowel disease
CS: Corticosteroids
CDAI: Crohn’s disease activity index
CRP: C-reactive protein
IBDQ: Inflammatory bowel disease questionnaire
CD-EIS: CD endoscopic index of severity
RCT: Randomized clinical trial
CMV: Cytomegalovirus
SC: Stem cell
SES-CD: Simplified endoscopy score for CD
