# Supplementary material for: Stem cell therapy for Crohn’s disease: systematic review and meta-analysis of preclinical and clinical studies
Source: Stem Cell Res Ther. 2021 Aug 18;12:463. doi: 10.1186/s13287-021-02533-0 (PMC8375136; doi:10.1186/s13287-021-02533-0)
Supplement: Supplementary file 1 — Additional file 1:Table S1: The study quality of animal studies; Figure S1: Publication bias of the outcomes: histopathological score, colon length, MPO activity, CDAI; Figure S2: The sensitivity analysis about DAI scores of the 1st day and the 3rd day after the treatment; Figure S3: The sensitivity analysis about DAI score of the 7th day and the 9th day after the treatment; Figure S4: The sensitivity analysis about colon length in the rat group; Figure S5: The subgroup analysis of histopathological score in the mouse group about modeling methods; Figure S6: The sensitivity analysis of histopathological score in the DSS mouse model group; Figure S7: The sensitivity analysis of histopathological score in the TNBS mouse model and the forest plot about the level of MPO activity of animal studies. Figure S8: The forest plot of IL-6, and IL-17 in the both mRNA and protein levels. Figure S9: The forest plot of IL-10 and TNF-α in the both mRNA and protein levels; Figure S10: The sensitivity analysis of HSC subgroup and subgroup analysis of CDAI score of different stem cell sources. Figure S11: The sensitivity analysis of CDAI scores of different treatment times; Figure S12: The subgroup analysis of IBDQ scores of different stem cell types, the forest plots of CRP and the adverse events happened in the experimental and placebo groups. [file 13287_2021_2533_MOESM1_ESM.docx]

**Supplement data**

**Table S1** SYRCLE’s RoB tool for each experimental animal studies.

|  | Random Sequence Generation | Baseline Characteristics | Allocation Concealment | Random Housing | Blinding (study Team) | Random Outcome Assessment | Blinding (Outcome Assessors) | Incomplete Outcome Data | Selective Outcome Reporting |
| --- | --- | --- | --- | --- | --- | --- | --- | --- | --- |
|  | SELECTION BIAS | | | PERFORMANCE BIAS | | DETECTION BIAS | | ATTRITION BIAS | REPORTING BIAS |
| Dorian Forte | ? | ? | - | + | ? | - | ? | + | + |
| Kang Chao | ? | ? | - | + | ? | - | ? | + | + |
| Jianguo Gao | ? | ? | - | + | ? | - | ? | + | + |
| Young Sun Nam | ? | ? | - | + | ? | - | ? | + | + |
| Fuyuan Yang | ? | ? | - | + | ? | - | ? | + | + |
| E Gonzalez-Rey | ? | ? | - | ? | ? | - | ? | + | + |
| Yan Lin | ? | ? | - | + | ? | - | ? | + | + |
| Antara Banerjee | ? | ? | - | + | ? | - | + | + | + |
| Woo Jin Song | ? | ? | - | + | ? | - | + | + | + |
| Ji Young Lim | ? | ? | - | ? | ? | - | ? | + | + |
| Shuichi Miyamoto | ? | ? | - | + | - | - | + | + | + |
| Shunzo Ikarashi | ? | + | - | + | - | - | ? | + | + |
| Ying Li | ? | ? | - | + | - | - | ? | + | + |
| Hyun Jung Lee | ? | ? | ? | + | ? | - | ? | + | + |
| Hannah Jo | ? | ? | - | + | ? | - | ? | + | + |
| Maryam Heidari | ? | ? | - | + | - | - | - | + | + |
| Yuzo Kawata | ? | ? | ? | + | - | - | ? | + | + |
| Yeonsil Yu | ? | ? | ? | + | - | + | + | + | + |
| Yousheng Lu | ? | ? | - | ? | ? | ? | ? | + | + |
| Hiroki Tanaka | ? | ? | ? | ? | ? | + | + | + | + |
| Xiaoyong Chen | ? | ? | - | ? | - | ? | ? | + | + |
| Ilse Molendijk | ? | ? | - | + | ? | + | + | + | + |
| Lu Liang | ? | ? | ? | + | ? | + | + | + | + |
| Patricia Fuenzalida | ? | ? | ? | + | ? | ? | ? | + | + |
| Liren Li | ? | ? | ? | + | ? | ? | ? | + | + |
| Fumio Tanaka | ? | ? | ? | + | ? | ? | ? | + | + |
| Aleksandar Nikolic | ? | ? | ? | + | + | + | + | + | + |
| Zhen Nan | ? | ? | ? | ? | ? | ? | ? | + | + |
| Minghao Xie | ? | ? | ? | ? | - | + | + | + | + |

+: low risk of bias - : high risk of bias ?: unclear risk of bias

**
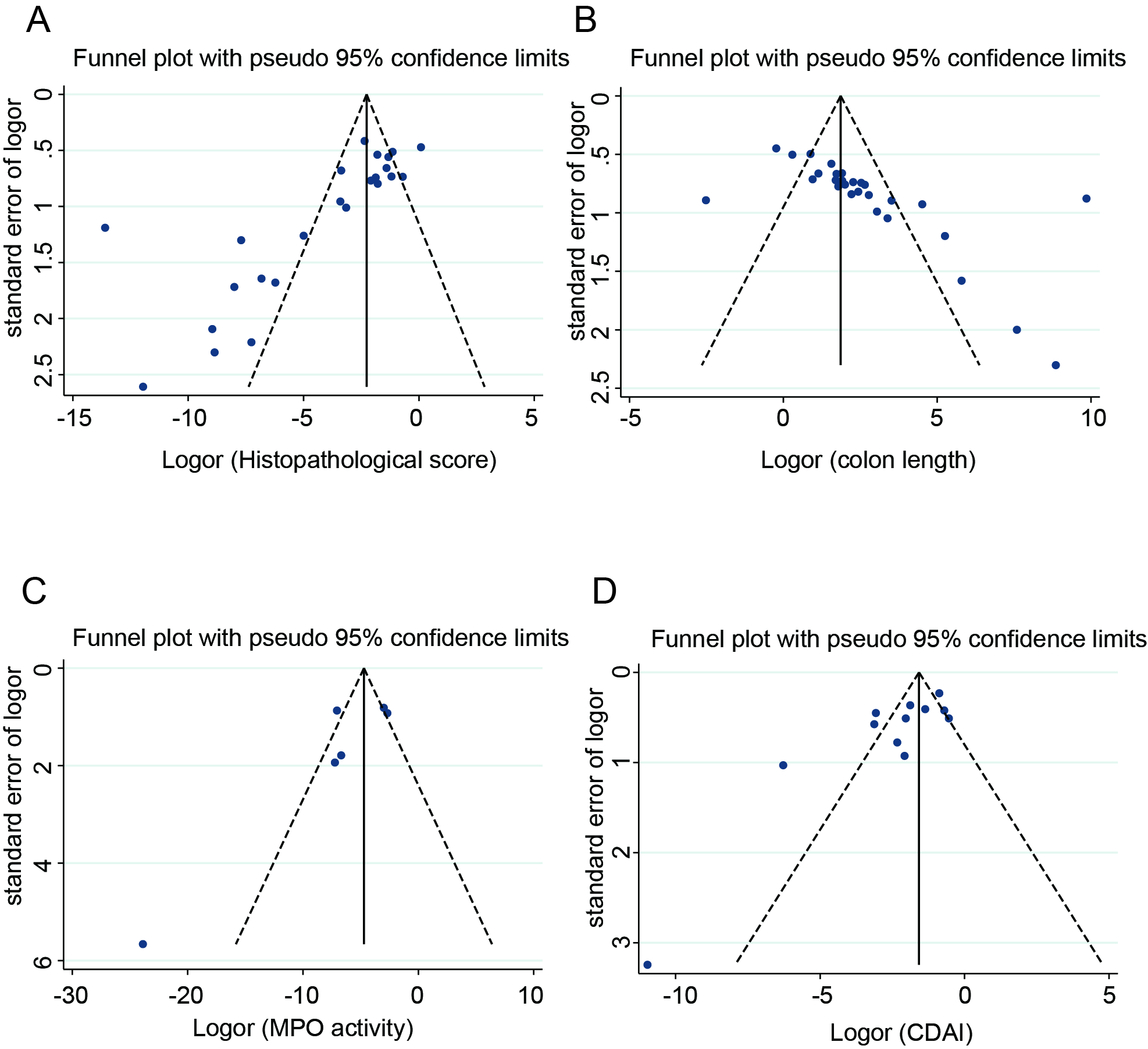
**

**Figure S1** Publication bias of the outcomes. Funnel plot (A-D): histopathological score, colon length, MPO activity, CDAI.

**
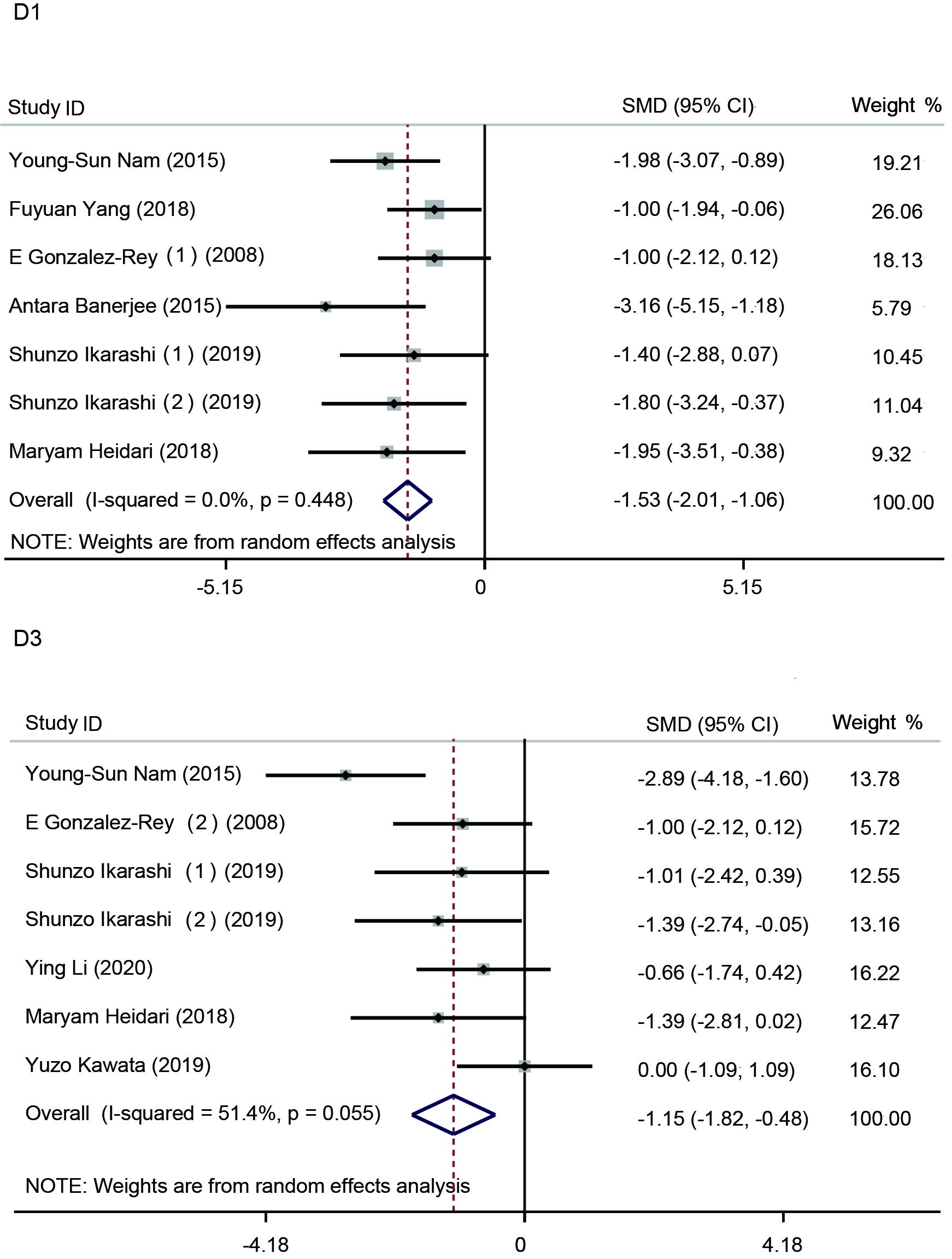
**

**Figure S2** The sensitivity analysis of each time subgroup about DAI expect the 5th day after the treatment. The sensitivity analysis about DAI score of the 1st day and the 3rd day after the treatment.


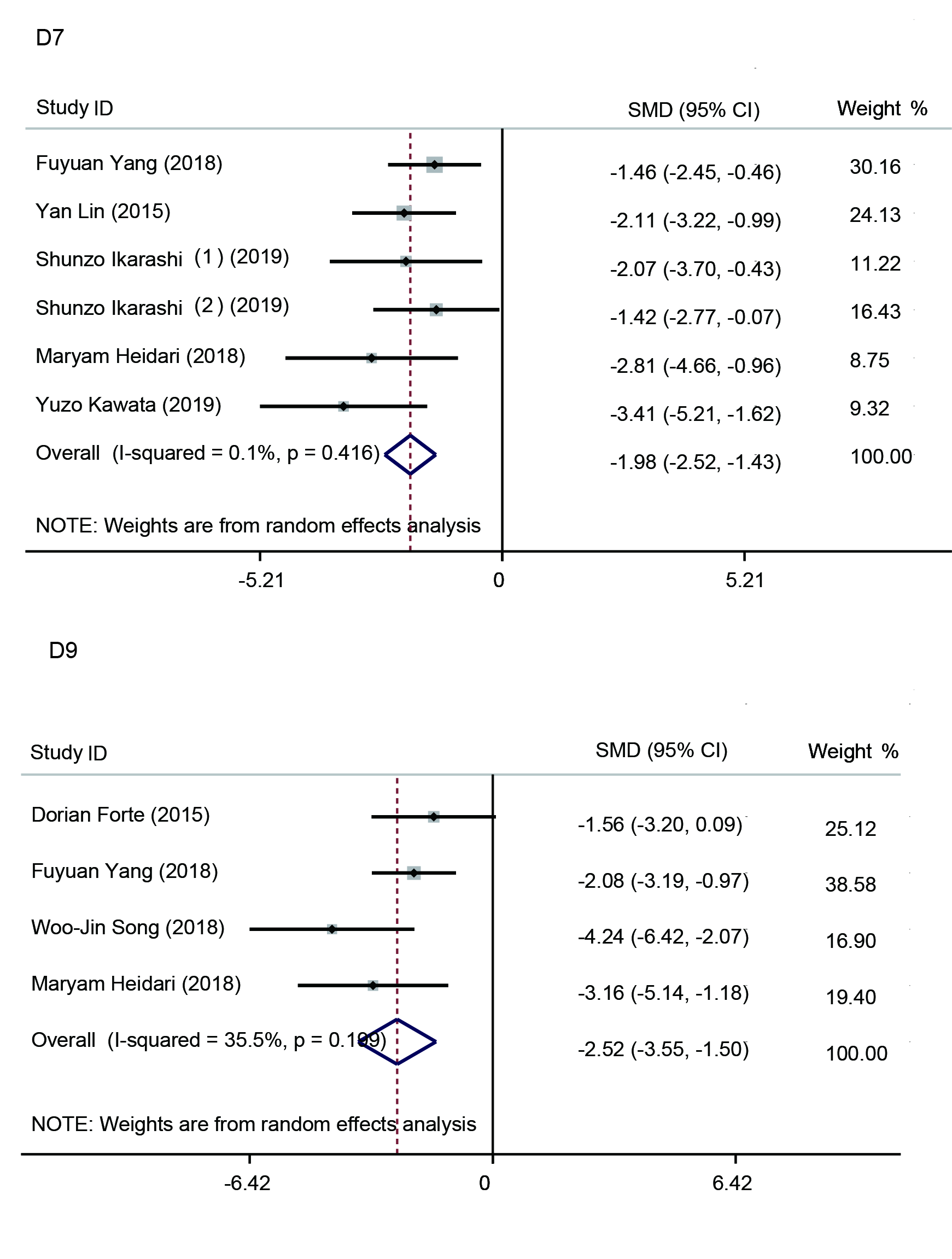


**Figure S3** The sensitivity analysis about DAI score of the 7th day and the 9th day after the treatment.


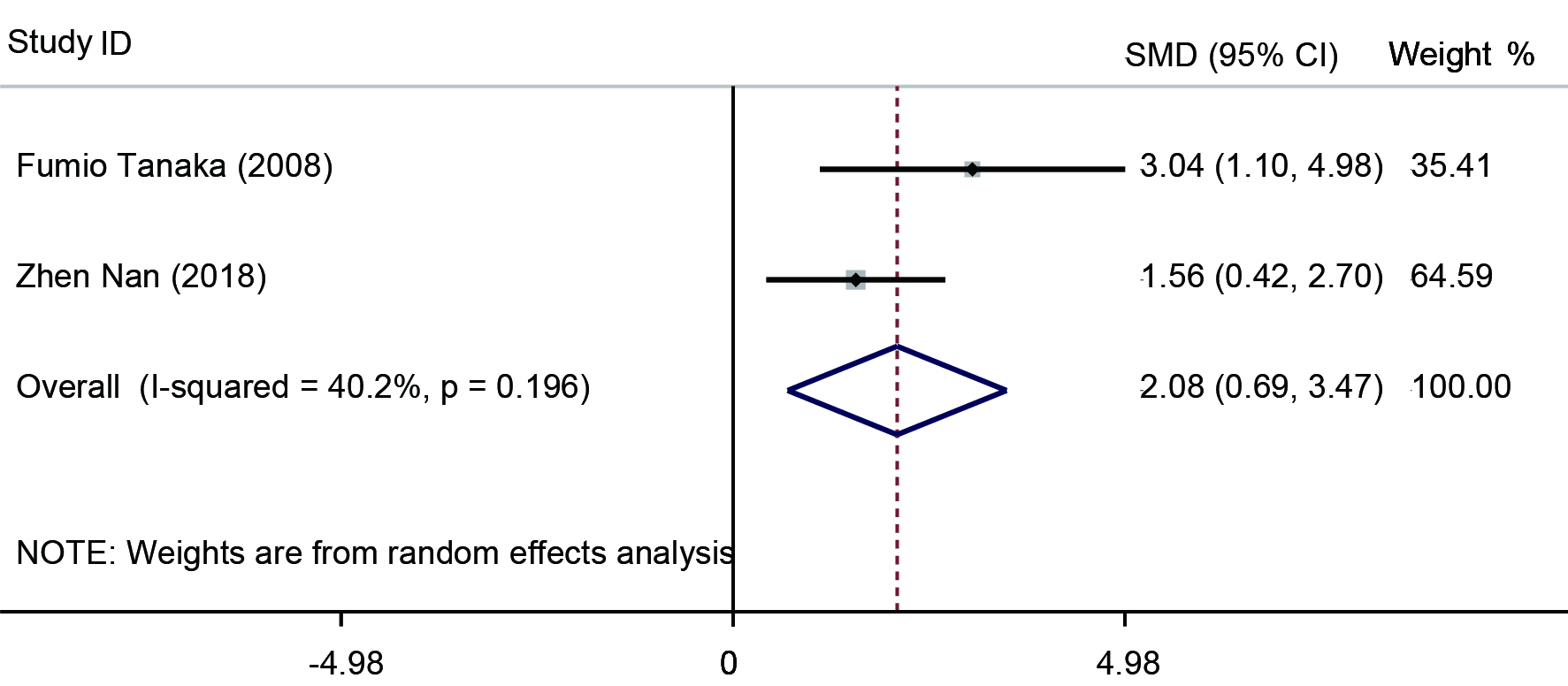


**Figure S4** The sensitivity analysis about colon length in the rat group.

**
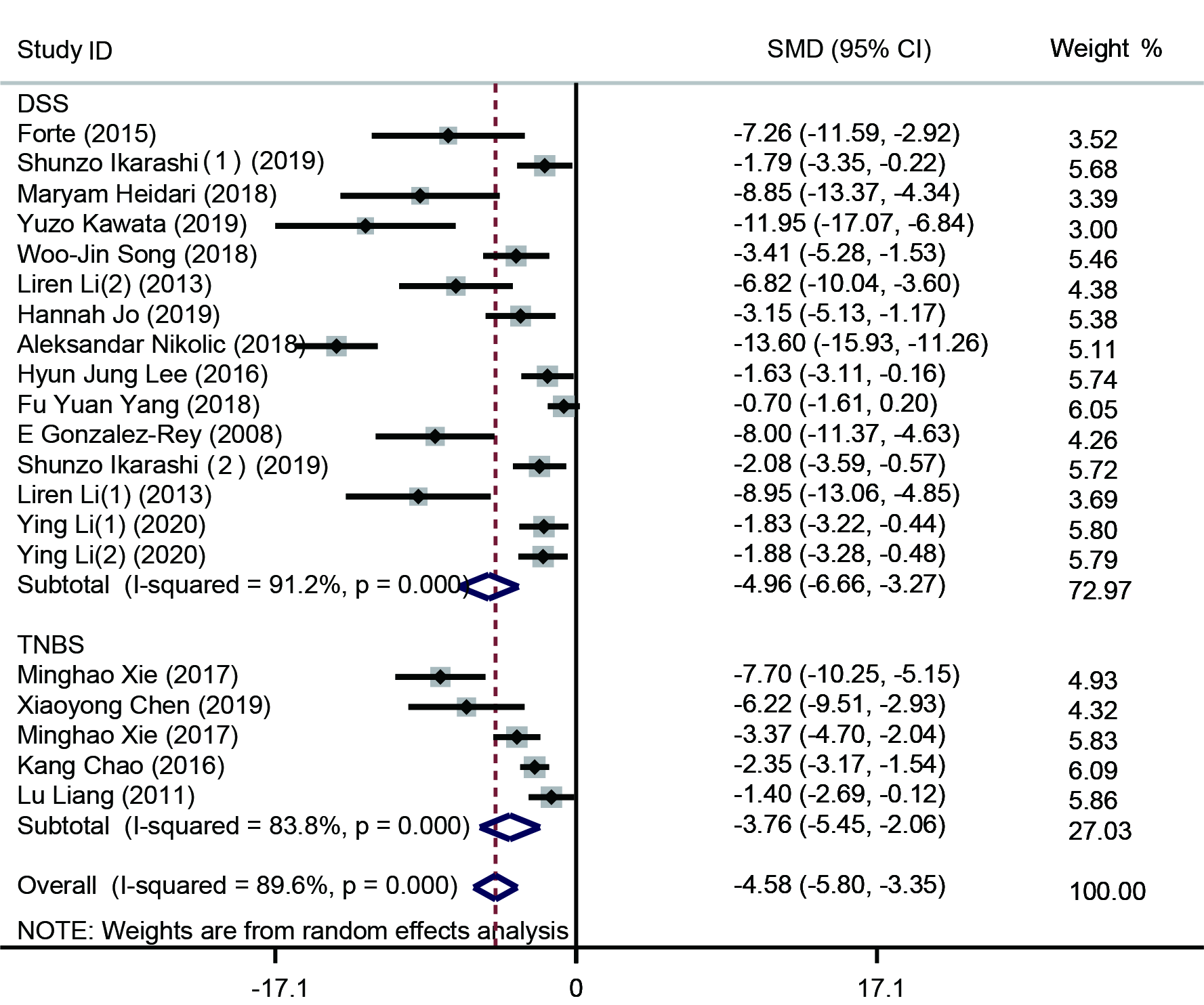
**

**Figure S5** The subgroup analysis of histopathological score in the mouse group about modeling methods.

**
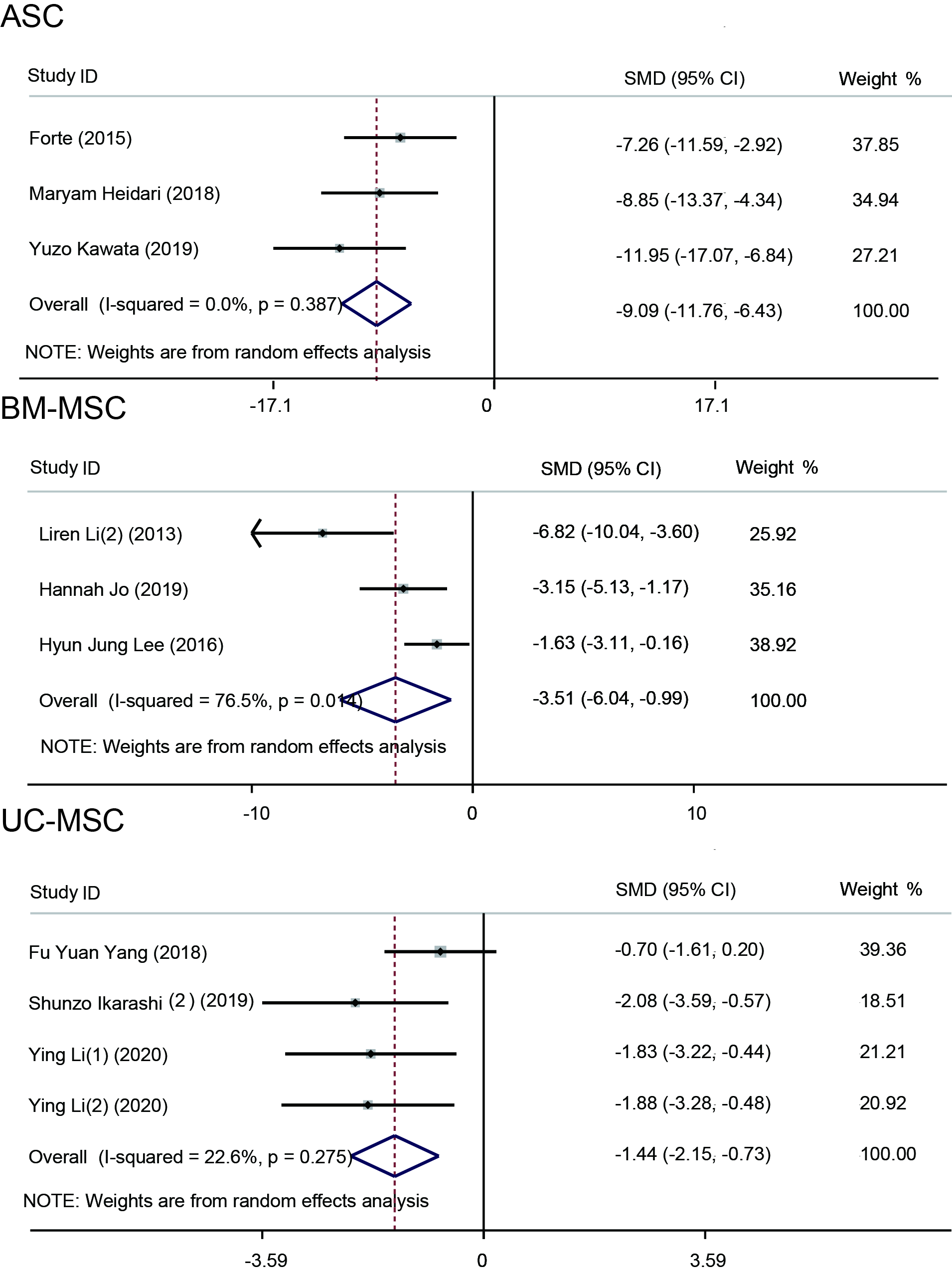
**

**Figure S6** The sensitivity analysis of histopathological score in the mouse group which modeled by DSS.


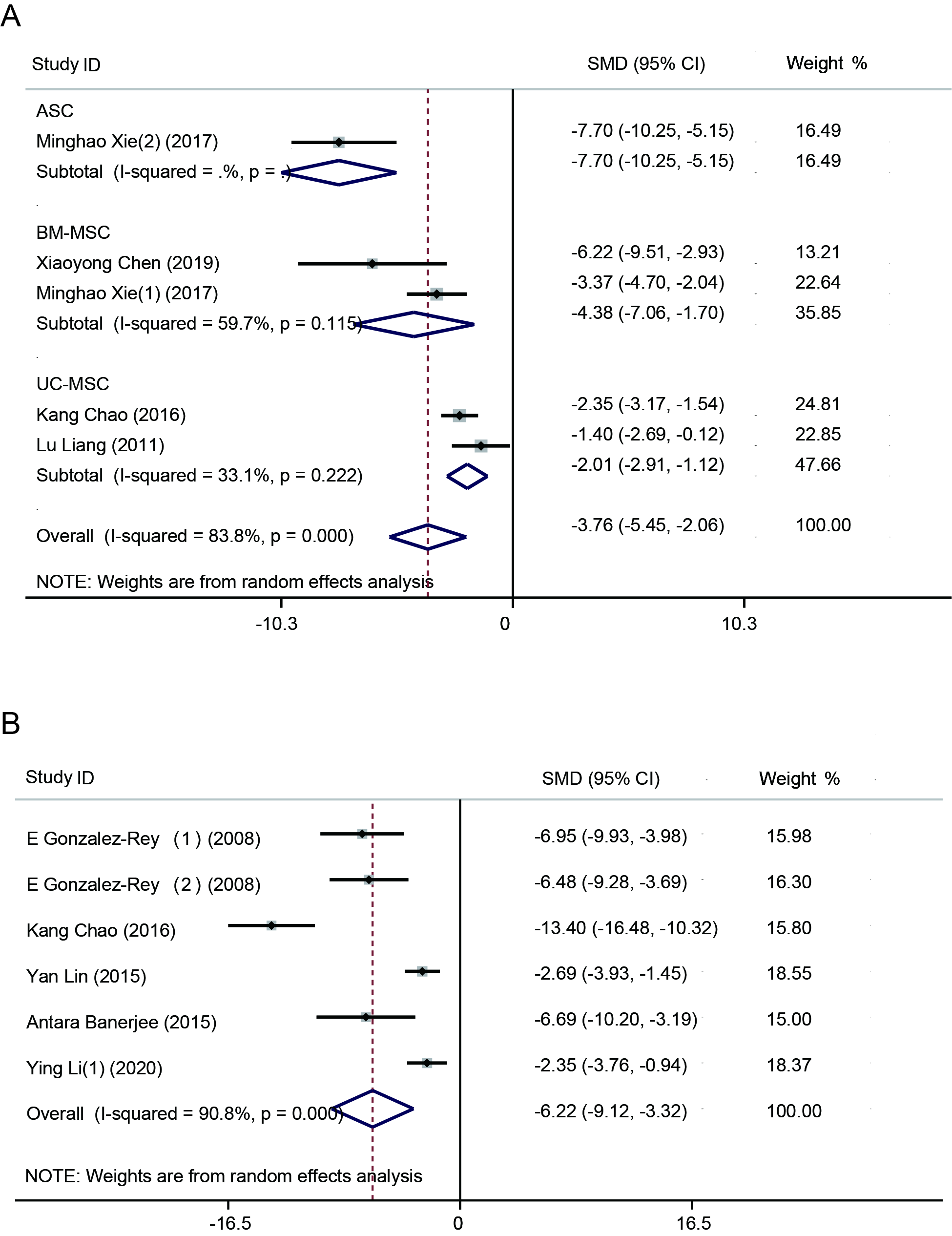


**Figure S7** (A)The sensitivity analysis of histopathological score in the mouse group which modeled by TNBS; (B)The forest plot about the level of MPO activity of animal studies.

**
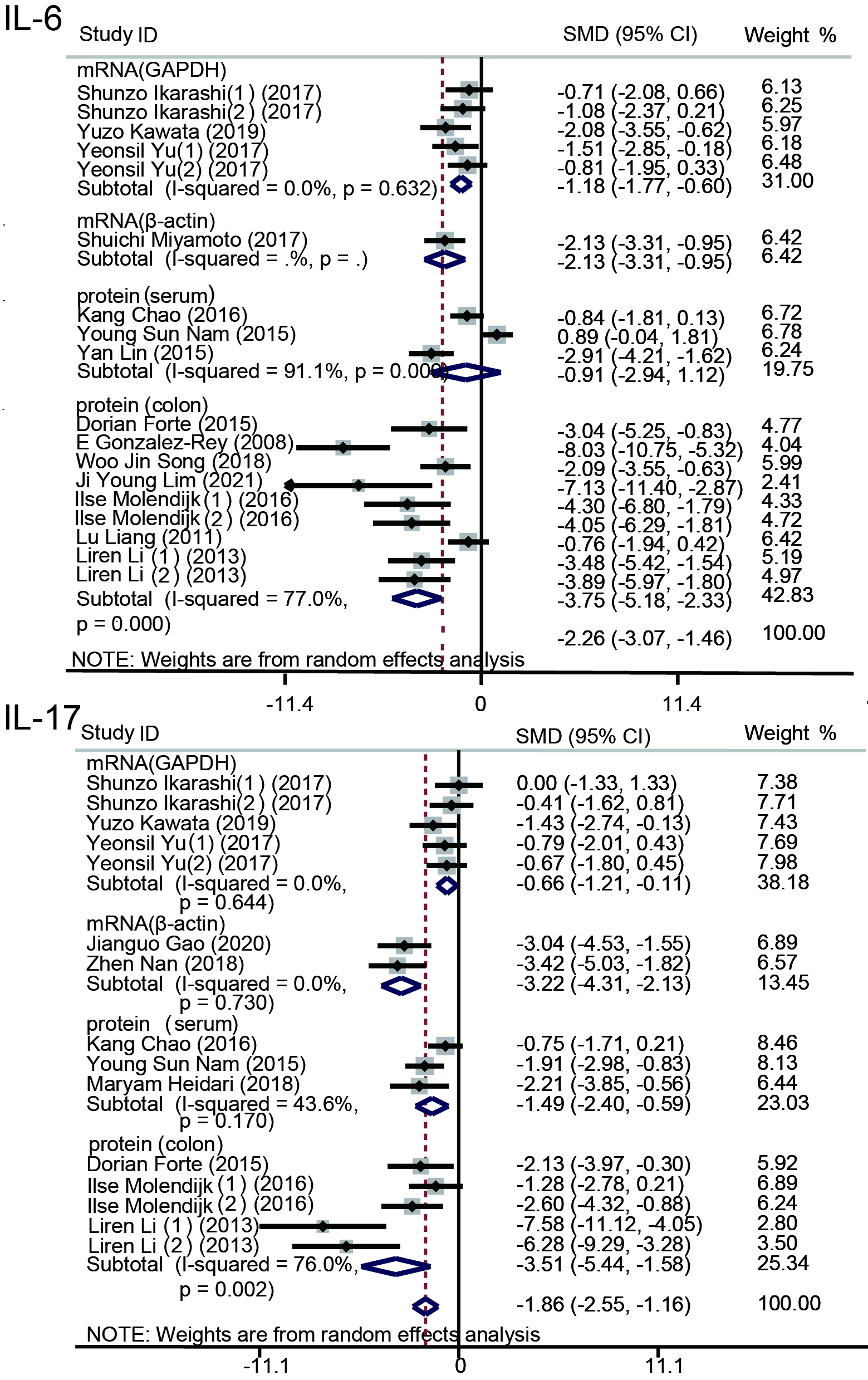
**

**Figure S8** The forest plot of IL-6, and IL-17 in the both mRNA and protein levels.

**
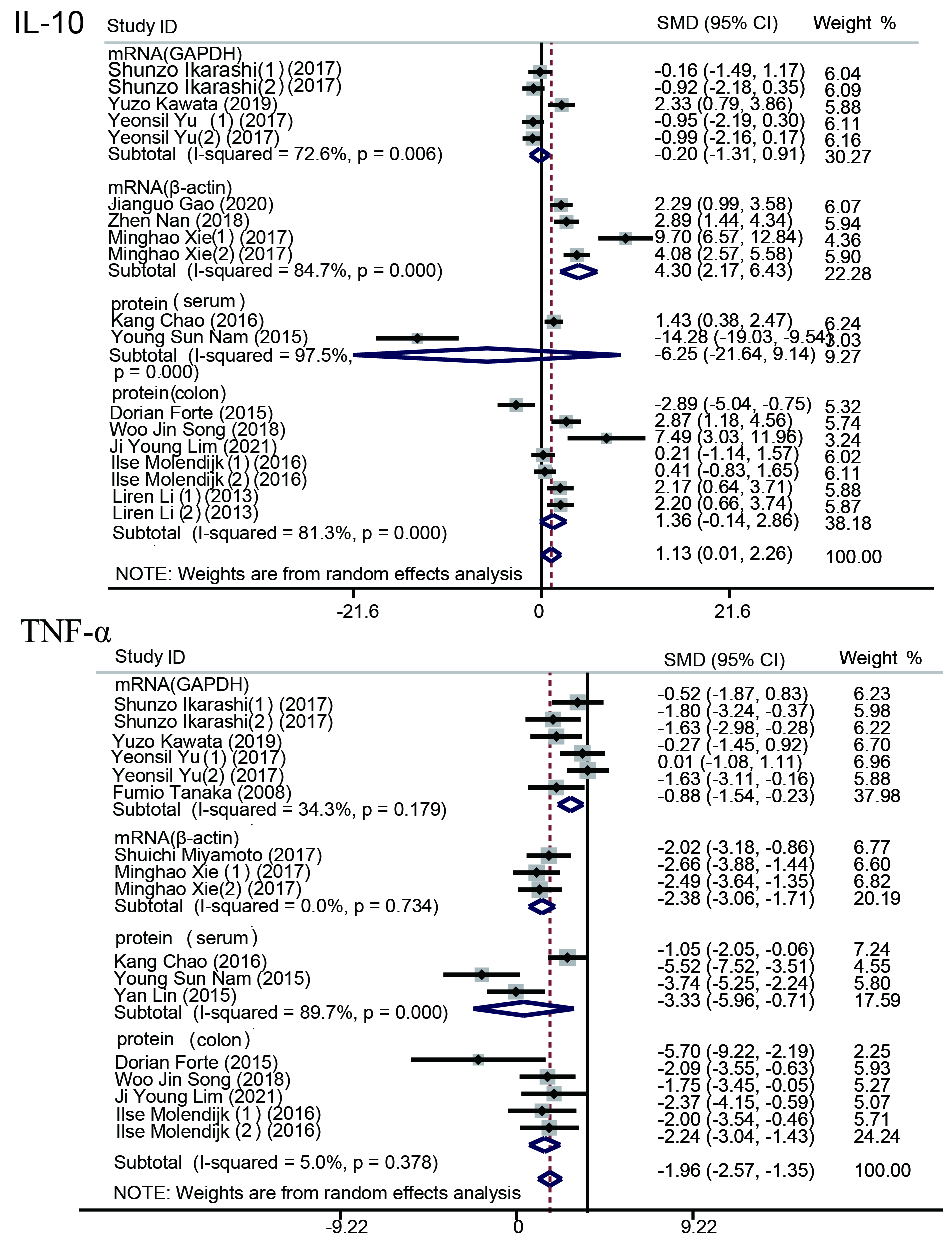
**

**Figure S9** The forest plot of IL-10 and TNF-α in the both mRNA and protein levels.

**
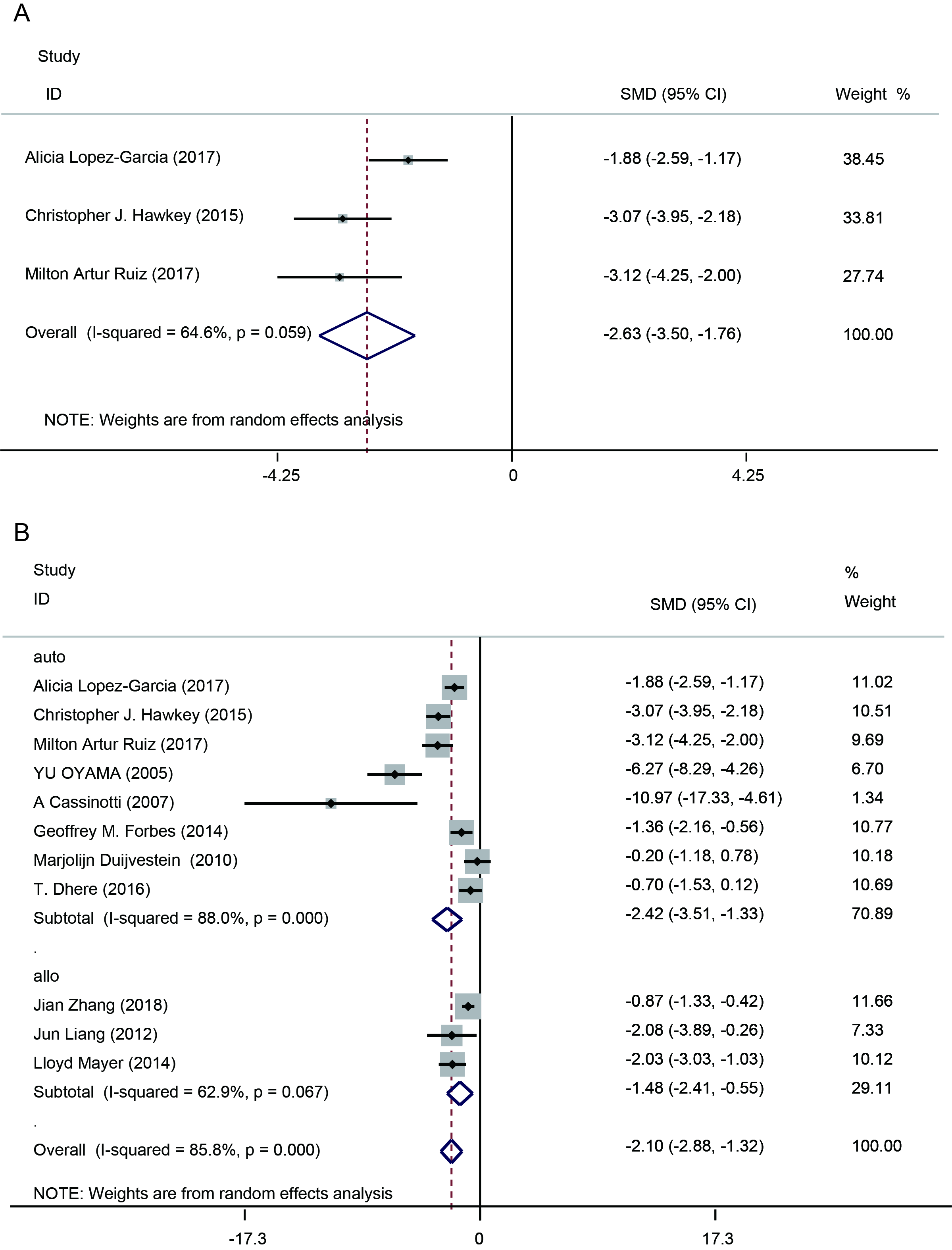
**

**Figure S10**  (A) The sensitivity analysis of HSC subgroup. (B)The subgroup analysis of CDAI score by the source of stem cells.


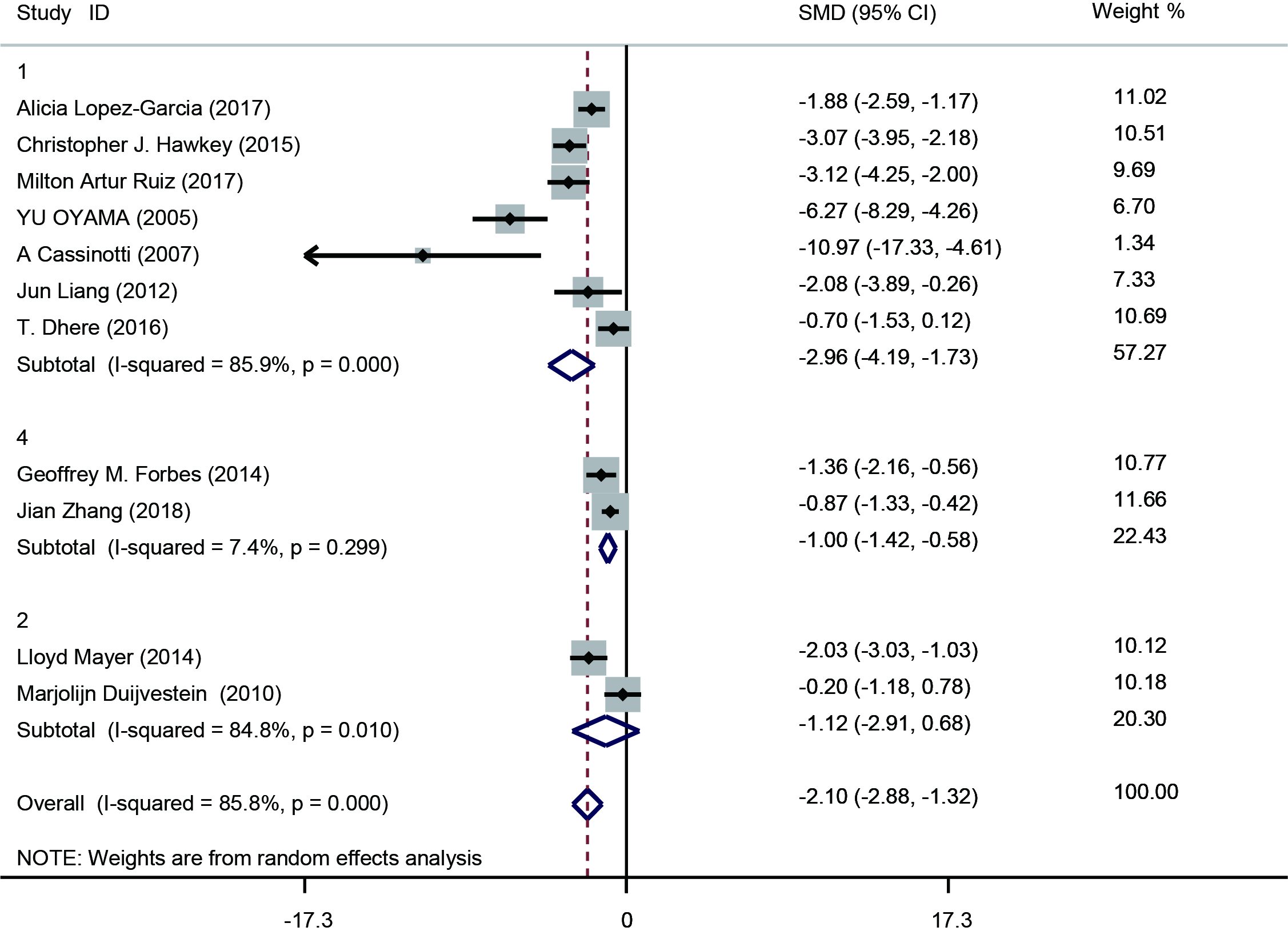


**Figure S11**  The sensitivity analysis of CDAI score by the treatment times.

**
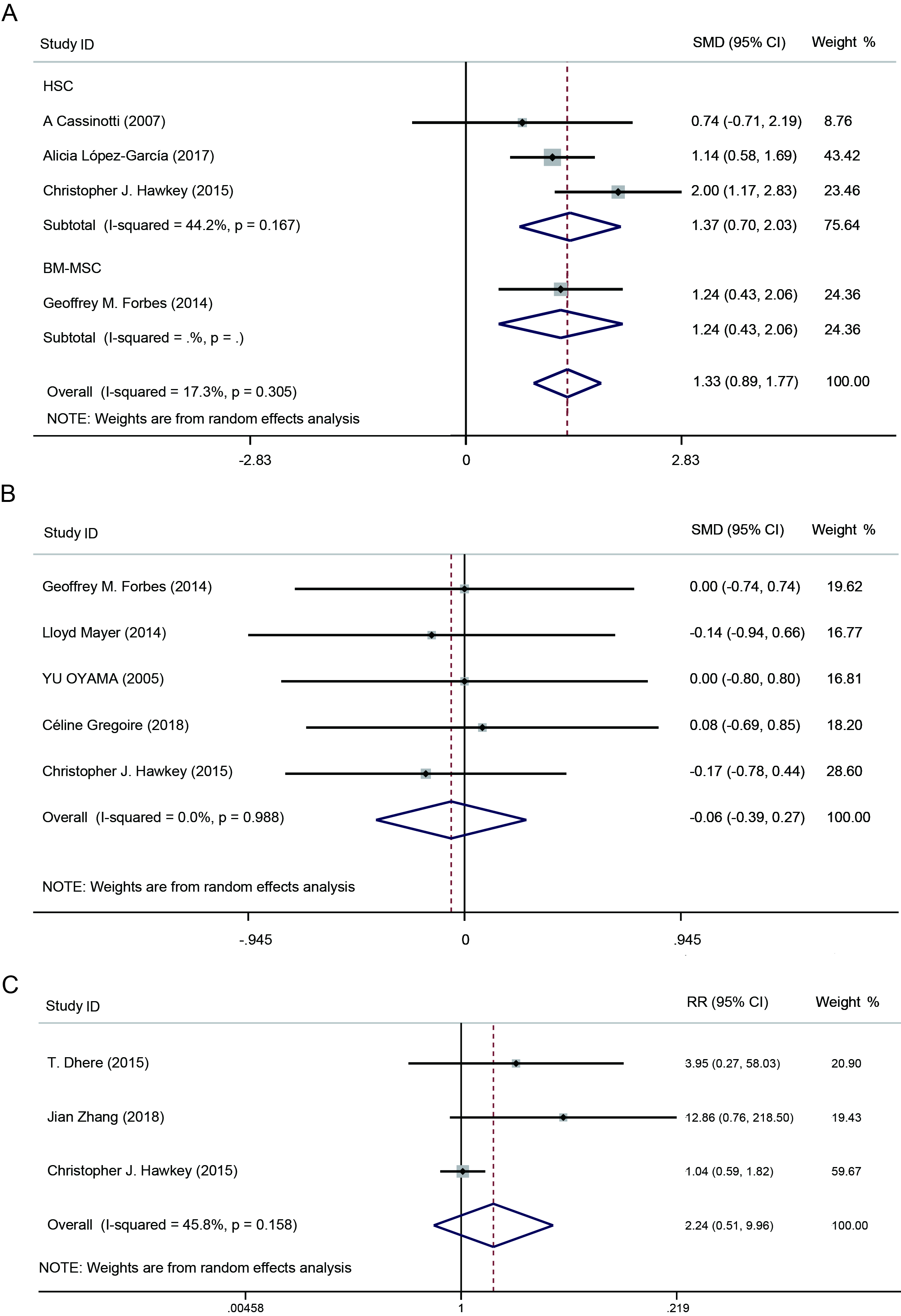
Figure S12** (A) The subgroup analysis of IBDQ score by the stem cell types. (B) The forest plot of CRP. (C) The forest plot of the adverse events happened in the experimental and placebo groups.
